# Structural basis of binding the unique N-terminal domain of microtubule-associated protein 2c to proteins regulating kinases of signaling pathways

**DOI:** 10.1016/j.jbc.2024.107551

**Published:** 2024-07-11

**Authors:** Viktor Bartošík, Jitka Plucarová, Alice Laníková, Zuzana Janáčková, Petr Padrta, Séverine Jansen, Vojtěch Vařečka, Tobias Gruber, Stephan M. Feller, Lukáš Žídek

**Affiliations:** 1 National Centre for Biomolecular Research, Faculty of Science, Masaryk University, Brno, Czech Republic; 2 Central European Institute of Technology, Masaryk University, Brno, Czech Republic; 3 Institute of Chemistry, Faculty of Science, Masaryk University, Brno, Czech Republic; 4 Institute of Molecular Medicine, Tumor Biology, Martin-Luther-University of Halle-Wittenberg, Germany; 5 Institute of Physics, Biophysics, Martin-Luther-University of Halle-Wittenberg, Germany

**Keywords:** microtubule associated protein (MAP) 2, protein kinase A (PKA), growth factor receptor-bound protein 2 (GRB2), A-kinase anchoring protein (AKAP), nuclear magnetic resonance (NMR)

## Abstract

Isoforms of microtubule-associated protein 2 (MAP2) differ from their homolog Tau in the sequence and interactions of the N-terminal region. Binding of the N-terminal region of MAP2c (N-MAP2c) to the dimerization/docking domains of the regulatory subunit RIIα of cAMP-dependent protein kinase (RIIDD_2_) and to the Src-homology domain 2 (SH2) of growth factor receptor-bound protein 2 (Grb2) have been described long time ago. However, the structural features of the complexes remained unknown due to the disordered nature of MAP2. Here, we provide structural description of the complexes. We have solved solution structure of N-MAP2c in complex with RIIDD_2_, confirming formation of an amphiphilic α-helix of MAP2c upon binding, defining orientation of the α-helix in the complex and showing that its binding register differs from previous predictions. Using chemical shift mapping, we characterized the binding interface of SH2-Grb2 and rat MAP2c phosphorylated by the tyrosine kinase Fyn in their complex and proposed a model explaining differences between SH2-Grb2 complexes with rat MAP2c and phosphopeptides with a Grb2-specific sequence. The results provide the structural basis of a potential role of MAP2 in regulating cAMP-dependent phosphorylation cascade *via* interactions with RIIDD_2_ and Ras signaling pathway *via* interactions with SH2-Grb2.

Microtubule-associated proteins (MAPs) regulate stability and dynamics of microtubules. MAP2 and Tau are MAPs expressed in brain neurons in several splicing variants. MAP2 and Tau differ in their cellular localization. MAP2 is found mainly in cell body and dendrites (1), whereas Tau is localized preferentially in axons (2). MAP2c is the shortest variant of MAP2, consisting of 467 amino acids (rat MAP2c). It is expressed prenatally and in brain regions exhibiting postnatal plasticity (3, 4). MAP2c consists of two domains. The N-terminal domain of MAP2c has a unique sequence, distinguishing MAP2c from Tau. The C-terminal domain has a high sequence homology with Tau isoforms (5). The MAP2c domains can be further divided to several regions, including N-terminal region, variable central region, proline-rich regions, microtubule-binding repeats (MTBRs), and C-terminal region (6). The domain organization of MAP2c is depicted in Figure 1. The microtubule-regulating function of MAP2 and Tau is associated with MTBRs. In spite of the high overall sequence homology of MTBRs, small variations in sequences result in very different pathological roles of MAP2 and Tau (7). Certain regions of Tau MTBRs tend to aggregate and form paired helical filaments and neurofibrillary tangles in the brains of patients suffering from the Alzheimer disease (8). MTBRs in MAP2 do not form such aggregates (7). Therefore, MAP2 are not involved in neurodegenerative diseases like Tau. Yet, MAP2 malfunction may result in pathological states such as depression (9) or schizophrenia (10, 11).

**Figure 1:**
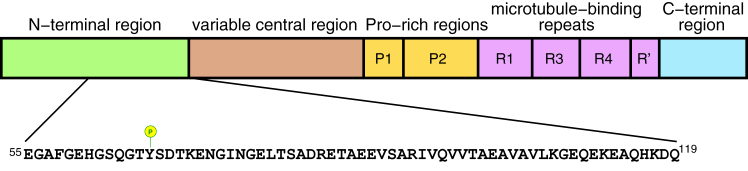
**Domain organization of MAP2c.** Sequence of the region investigated in this study is displayed below the diagram. Tyrosine phosphorylated by Fyn is labeled.

As mentioned above, the major differences between MAP2 and Tau can be found in the N-terminal domain. In this study, we explore interactions of the first 159 residues of MAP2c (cf Fig. 1). Deletion of this region results in the disruption of hippocampal neuron architecture and deficits in contextual memory in mice (12). The N-terminal region interacts with binding partners involved in functions other than direct microtubule regulation. In particular, interactions with the type II regulatory subunit (RII) of cAMP-dependent protein kinase PKA and with growth factor receptor-bound protein 2 (Grb2) attracted great attention. PKA and Grb2 control the phosphorylation of multiple proteins downstream the signaling pathways they are involved in. The functions of PKA and Grb2 are regulated by binding various proteins, including MAP2.

PKA is a ubiquitous kinase playing important roles in brain neurons (13). In its inactive form, PKA is present as a tetrameric holoenzyme consisting of two regulatory (R) and two catalytic (C) subunits (14). The specificity of PKA signaling is achieved by expressing R subunits as nonredundant isoforms of two types (RI and RII), each having two subtypes (RIα, RIβ, RIIα, and RIIβ). The enzyme is localized in the cell by interactions of the R subunits with A-kinase anchoring proteins (AKAPs) tethered to the plasma membrane, cytoskeleton, or various organelles (15). Most AKAPs bind RII subunits preferentially (16), but dual-specific (17, 18) and RI-specific (19, 20, 21, 22) AKAPs have been also described. Binding of cAMP to the R subunits activates PKA by releasing the C subunits that phosphorylate their target proteins in the cellular compartment determined by the AKAP anchoring. All AKAPs contain a relatively long amphipathic α-helix that tightly binds the dimerization/docking domains of the R subunits (23). Solution and crystal structures of the dimerization/docking domains with the AKAP helices confirmed that the interactions are primarily hydrophobic (23, 24, 25, 26), while electrostatic interactions seem to define the specificity (23). Based on the available structures and on sequence alignment of AKAPs, a model explaining the AKAP specificity and predicting the register of interacting AKAP residues was proposed (23).

MAP2 isoforms, anchoring PKA to microtubules, were the first AKAPs identified (27), binding preferentially the RIIα subunit of PKA (RIIα-PKA) (28, 29). Experiments with successively smaller MAP2 fragment identified that MAP2 residues Asp83–Glu113 are sufficient for the binding (30). The MAP2:RII interaction plays a major role in controlling the PKA activity in the dendrites of hippocampal and cortical pyramidal neurons (13, 31). It was reported that MAP2 is the dominant AKAP in pyramidal dendrites, restricting more than 97% of the RII subunits to dendritic shafts (13). Anchoring of PKA by MAP2 is an important mechanism of the regulation of postsynaptic functions (13) and is involved in the antidepressant response (32).

PKA anchoring by MAP2 also influences the function of MAP2 itself. MAP2 isoforms act both as upstream regulators and downstream targets of cAMP-dependent pathways. Activity of PKA modulates microtubule bundling by MAP2 (33). Earlier, we have studied MAP2c as a substrate of PKA, in comparison with its homolog Tau. PKA phosphorylates MAP2c outside MTBRs. PKA phosphorylation of MAP2c thus does not directly interfere with microtubule binding, in contrast to the inhibiting effect of PKA phosphorylation on Tau–microtubule interactions (34). However, PKA phosphorylation reduces MAP2c interactions with microtubules indirectly, as it enhances the binding of MAP2c to the regulatory protein 14-3-3, which competes with the MAP2c–microtubule interactions (34).

Grb2 was first described as a linker coupling growth factor receptor tyrosine kinases (RTKs) to the rat sarcoma virus (Ras) signaling pathways (35). Grb2 structure (36) consists of two Src-homology 3 (SH3) domains, recognizing PXXP motifs, and of one Src-homology 2 (SH2) domain, binding phosphorylated tyrosine. The Grb2 SH2 domain (Grb2-SH2) interacts with phosphorylated RTKs, and the SH3 domains bind the mammalian homologs of *Drosophila Son of sevenless* (SOS) gene products. The interaction of Grb2 with phosphorylated RTKs triggers the guanine nucleotide exchanging activity of SOS. The activated SOS then releases GDP from Ras, which can bind GTP and activate the Ras signaling pathway (36, 37, 38, 39).

The described function of Grb2 is modulated by interactions with additional proteins, including MAP2. Specific phosphorylation by kinase Fyn at Tyr67 of human MAP2c and subsequent binding to the Grb2 SH2 domain was reported (39). A detailed study of structure and dynamics of the Grb2-SH2 in complex with a synthetic peptide (not derived from MAP2c) was published recently (40). Interestingly, Asn69, determining the specificity of binding to Grb2-SH2, is often replaced by other amino acids in otherwise highly conserved mammalian MAP2c sequences. The MAP2c sequence also includes one class I SH3-binding site (among 13 PXXP motifs) that interacts with Grb2 (37, 39, 41). Binding to the SH3 domain does not require specific phosphorylation, but it is modulated by the activity of protein kinases. For example, MAP2c is phosphorylated by extracellular signal-regulated kinase 2 (ERK2) of the Ras pathway (41). Interestingly, ERK2 phosphorylation interferes with the MAP2c binding to Grb2, which may represent a feedback in the MAP2c–Grb2 interactions (37, 41). We can therefore assume that MAP2c interferes in a phosphorylation-dependent manner with the Grb2 function at two levels: (i) by competing with the RTKs, but also phosphatases (42, 43) for the SH2 domain and (ii) by competing with SOS (and other Grb2-binding proteins) for the SH3 domain. In this regulatory network, interactions of MAP2c with SH2 and SH3 domains of Grb2 are controlled by different kinases. Binding to the SH2 domain is activated by nonreceptor tyrosine kinase Fyn, whereas binding to the SH3 domain is suppressed by proline-directed Ser/Thr kinase ERK2 (37, 41).

MAP2 and Fyn are involved in neuronal morphogenesis and migration (12, 31, 44, 45, 46). Grb2 is ubiquitously expressed with developing brain being not an exception (39, 47). Grb2 was reported to play role in neurite outgrowth and branching in cultured mouse embryonic cortical neurons (48). Tyrosine-phosphorylated MAP2c and its interaction with Grb2 was detected in human fetal brain (39). On the other hand, tyrosine phosphorylation was not detected in MAP2 from neonatal or adult rat brains (38). Tyrosine phosphorylation of putative MAP2c was also observed in fetal primary neuronal cultures. Fyn was found to be highly active in the fetal neuronal culture, and the tyrosine phosphorylation of putative MAP2c was diminished by the inhibitor of Fyn and other Src family tyrosine kinases (49). Aforementioned findings imply that tyrosine phosphorylation of MAP2c and subsequent interaction with the SH2 domain of Grb2 might be involved in prenatal brain development. Besides the proposed physiological role, MAP2–Grb2 interaction might be of interest in glioma pathophysiology. It was reported that MAP2 interacts with Grb2 in glioma cells and that downregulation of MAP2 promotes the interaction between Grb2 and SOS. Moreover, miR-484, that is associated with poor prognosis in glioma, targets MAP2. It was suggested that MAP2 downregulation *via* miR-484 enhances Grb2–SOS interaction and subsequently upregulates ERK signaling pathway which leads to enhanced stemness of glioma cells (50).

As mentioned above, interactions of MAP2c with RIIα-PKA and Grb2 have been characterized biochemically already in 1982 (27) and 2001 (37), respectively. However, structural details of the complexes of MAP2c with RIIα-PKA or Grb2 have not been described yet. In this study, we addressed the following open questions: What is the structure of the complex of MAP2c with RIIα-PKA? Is it possible to determine the structure of the complex using the whole N-terminal MAP2c region? Do all MAP2c residues involved in the RIIα-PKA binding form a regular α-helix, found in other AKAP:RIIα-PKA complexes? Is it possible to predict the exact length of the interacting helix by sequence-based computational tools? How is the MAP2c helix oriented in the complex? Does the register of the MAP2c residues interacting with RIIα-PKA correspond to the proposed model of RII *versus* RI-interacting AKAP residues (23)? Does the rat MAP2c phosphorylated at Tyr67 interact with Grb2-SH2 despite of the consensus motif variation (replacement of Asn69 by aspartate)? If so, what is the structural consequence of the sequence variation? In order to answer the listed questions, we applied various NMR approaches to solve the solution structure of the dimeric RIIα-PKA dimerization/docking domain (RIIDD_2_) bound to the N-terminal region of MAP2c and to characterize the binding of rat MAP2c to Grb2.

## Results

### RIIα-PKA and its dimerization/docking domain binds to the N-terminal region of MAP2c with nanomolar affinity

**Figure 2:**
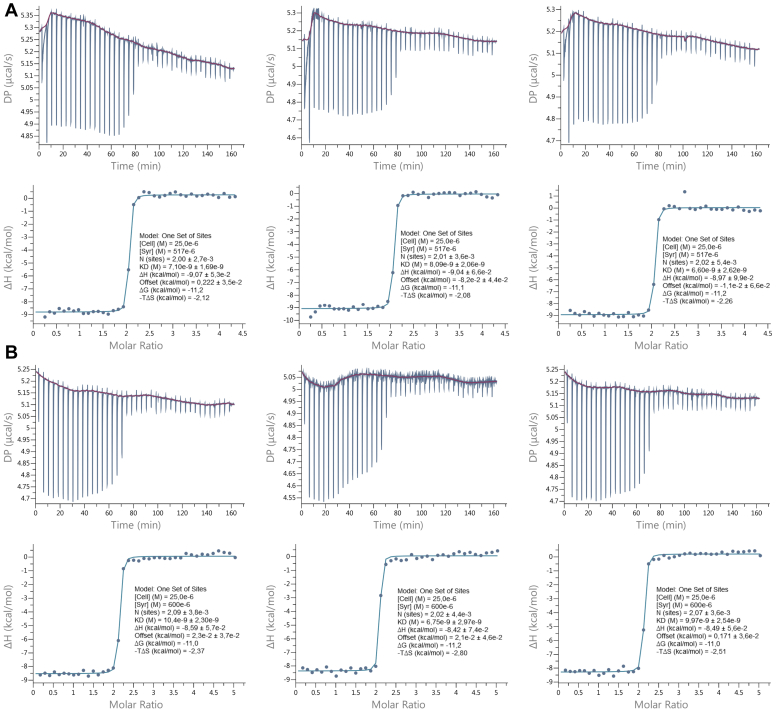
**ITC analysis of N-MAP2c binding to RIIα-PKA and RIIDD**_**2**_**.** Results of triplicate ITC measurements of the complex of N-MAP2c with RIIα-PKA (*A*) and RIIDD_2_ (*B*).

Isothermal titration calorimetry (ITC) of MAP2c and hexahistidine-tagged N-terminal fragment of MAP2c containing residues 1 to 159 (N-MAP2c) titrated by RIIα-PKA and RIIDD_2_ provided steep-binding isotherms corresponding to the dissociation constants *K*_D_ in a lower nanomolar range. Similar binding isotherms for MAP2c and N-MAP2c show that the main interaction site of RIIα-PKA is located in the N-terminal part of MAP2c. Thermograms, binding isotherms, and fitted values of triplicate experiments for N-MAP2c titrated by RIIα-PKA and RIIDD_2_ are presented in Figure 2, *A* and *B*, respectively. The results allowed us to determine the *K*_D_ of (7.3 ± 0.8) nM for the N-MAP2c:RIIα-PKA complex and *K*_D_ of (9.0 ± 2.0) nM for the N-MAP2c:RIIDD_2_ complex. The uncertainties, representing SDs of the triplicates, indicate that the data reliably define the order of magnitudes of *K*_D_ values, but their precision is limited by the relatively high ratio of the cell concentration to *K*_D_ (*c*/*K*_D_). In general, precise determination of *K*_D_ is challenging for *c*/*K*_D_ > 1000 and impossible for *c*/*K*_D_ > 10,000. Precision of our *K*_D_ values is in a good agreement with the obtained *c*/*K*_D_ ratio of ∼2500. On the other hand, the enthalpy Δ*H* = −(36.4 ± 2.1) kJ/mol and Δ*H* = −(35.6 ± 0.4) kJ/mol, respectively, and the stoichiometry 1:2 (N-MAP2c:RIIDD_2_ monomer) are determined very precisely. Similar data were obtained for MAP2c titrated by RIIα-PKA and RIIDD_2_ (Fig. S1, *A* and *B*, respectively, in Supporting information). However, quantitative determination of *K*_D_ was not possible for the complexes of MAP2c.

An estimate of *K*_D_ for the N-MAP2c:RIIDD_2_ complex can be obtained from the literature. Slot-blot overlay showed that RIIα-PKA binds human thyroid anchoring protein Ht31 with ∼1.9 times higher affinity than a peptide consisting of MAP2 residues 1 to 154 (51). For the Ht31:RIIα-PKA complex, *K*_D_ = 2.2 nM was determined by fluorescence polarization (52). It suggests *K*_D_ of ∼4 nM for the N-MAP2c:RIIα-PKA complex, which is in a good agreement with our ITC data.

In conclusion, ITC confirmed the expected strong binding of RIIα-PKA to the N-terminal domain of MAP2c. The estimated nanomolar affinities showed that N-MAP2c and RIIDD_2_ should form a sufficiently stable complex for NMR structural analysis.

### NMR peak broadening revealed MAP2c residues involved in RIIα-PKA binding

NMR peak broadening was used to map the residues of MAP2c influenced by the interactions with RIIα-PKA. Peaks of intrinsically disordered residues of MAP2c are sharp due to its fast motions in spite of its large size. The MAP2c residues interacting with RIIα-PKA become a part of a large, well ordered complex and their peaks broaden dramatically. Decrease in the peak height due to the broadening is thus a sensitive indicator of the interaction.

**Figure 3:**
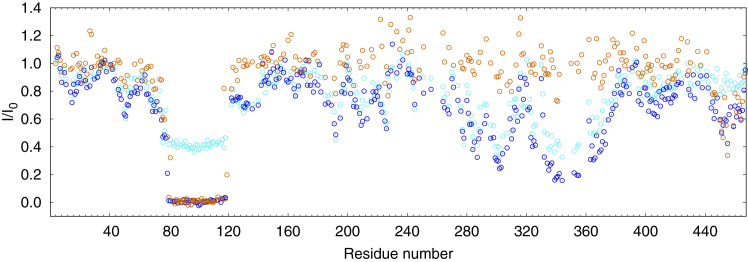
**Reduction of peak heights in the HNCO spectra of 100 μM [**^**13**^**C,**^**15**^**N]-MAP2c in the presence of 100 μM RIIα-PKA (*cyan*), 200 μM RIIα-PKA (*blue*), and 200 μM RIIDD**_**2**_**(*orange*, monomeric concentration).** The plotted *I*/*I*_0_ values are ratios of the peak heights of the MAP2c complexes to the peak heights of free MAP2c.

3D HNCO spectra (53) of [^13^C,^15^N]-MAP2c in a free form and in the complexes with RIIα-PKA and RIIDD_2_ were recorded, and peak heights measured (Fig. 3). Drop of the heights of peaks of [^13^C,^15^N]-MAP2c in the complexes indicated which MAP2c residues are involved in the interactions. Binding to RIIDD_2_ decreased peak heights almost exclusively between residues Thr80 and Asp118. The complex with RIIα-PKA exhibited a dramatic decrease in the same region of MAP2c, but a substantial broadening was also observed in other regions, especially in the proline-rich region P2 (residues Ser280–Leu300) and in the microtubule-binding repeat 3 (Val333–Arg363). It suggests weaker interactions of the C-terminal regions of MAP2c with other domains of RIIα-PKA. These weak interactions may contribute to the overall affinity of MAP2c for RIIα-PKA. In addition, we observed peak broadening in the C-terminal region of MAP2c in the presence of both RIIDD_2_ and RIIα-PKA. The C-terminal sequence _453_TLAEDVTAALAKQGL_467_ exhibits a high propensity to form a helical structure in free MAP2c (6, 34). As the α-helical conformation is a typical signature AKAPs, we used truncated MAP2c constructs to check how strongly the C-terminal MAP2c helix binds to RIIα-PKA. Results of ITC and NMR experiments present in Fig. S2 in Supporting Information show that the interaction is rather weak. *K*_D_ values of ∼30 μM and of ∼0.9 mM were measured for the MAP2c fragments consisting of residues 159 to 467 and 300 to 467, respectively. Therefore, we do not expect the C-terminal helix or other regions outside the N-terminal domain of MAP2c to compete significantly with residues Thr80–Asp118 for the RIIα-PKA binding.

In summary, we confirmed that MAP2c binds RIIα-PKA *via* the residues Thr80–Asp118, in agreement with the literature (24). Interactions with other regions of MAP2c were also observed, but their contribution to the overall binding was weak.

### NMR assignment was obtained for most N-MAP2c residues forming complex with RIIDD_2_

The peak broadening in the HNCO spectra documented that RIIα-PKA and RIIDD_2_ recognize the same major binding site in MAP2c. The calorimetric data showed that N-MAP2c and RIIDD_2_ are sufficient to form a stable complex. Therefore, these shorter constructs were used for the structure determination of the complex in order to reduce complexity of the NMR spectra.

Chemical shifts of the MAP2c residues in the complex were assigned using [^13^C,^15^N]-N-MAP2c and unlabeled RIIDD_2_ mixed in the 1:4 M ratio (expressed in concentration of the monomeric units of RIIDD_2_ forming a dimer). The ^1^H-^15^N heteronuclear single-quantum coherence (HSQC) spectrum (54, 55) showed sharp peaks with a narrow ^1^H frequency distribution, typical for disordered proteins, but also broader peaks spanning a larger range of ^1^H frequencies, revealing the presence of a well-ordered MAP2c region. Using the triple resonance HNCACB (56), CBCA(CO)NH (57), HNCA (53), and HN(CO)CA (58) spectra, backbone chemical shifts of disordered residues outside the RIIDD_2_ binding site were assigned.

Sensitivity of the triple resonance experiments was low for the residues of the RIIDD_2_-binding site, presumably due to a combination of a higher correlation time (due to the more rigid structure) and conformational exchange in the complex. For this reason, we decided to use residue-specific isotope labeling. The labeling strategy was based on a high occurrence of aliphatic amino acids in the N-terminal region of MAP2c. Valine and isoleucine were labeled with ^13^C and ^15^N, leucine only with ^15^N. Assignment of Val, Ile, and Leu residues relied mostly on HNCA, HN(CO)CA, ^15^N- and ^13^C-edited NOESY (59, 60, 61) spectra, their side-chains were assigned also using HC(C)H-TOCSY (62, 63) spectra of uniformly and residue-specifically labeled samples. In combination with the uniformly labeled samples, we were able to assign all residues bound to RIIDD_2_, except for Lys112, Lys117, Asp118, and Gln119. It should be noted that the assignment was facilitated by the presence of medium-range and intermolecular nuclear Overhauser effect (NOE). No medium-range or intermolecular NOE was observed for residues following Gln110. On one hand, this explains missing the assignment of Lys112 and residues 117 to 119. On the other hand, it documents that the missing assignment in this region does not result in a loss of information about intermolecular contacts in the complex.

### Complete NMR assignment of RIIDD_2_ in complex with N-MAP2c revealed breaking symmetry of RIIDD_2_ upon N-MAP2c binding

Free [^13^C,^15^N]-RIIDD_2_ was mixed with unlabeled N-MAP2c in 2:1 M ratio (expressed for the monomeric units of RIIDD_2_). The resulting ^1^H-^15^N HSQC spectrum showed 83 peaks, of which 64 were of backbone and 19 of side-chains. In the regions of Ile3–Ile5, Leu9–Gly15, Val18–Gln24, Ala42, and Ala44, two sets of chemical shifts were observed. It indicated that the *C*2 symmetry of RIIDD_2_ is broken by the interaction with N-MAP2c. All backbone chemical shifts were assigned using the triple resonance HNCACB (56), CBCA(CO)NH (57), HNCA (53), and HN(CO)CA (58) spectra. Side-chain ^1^H and ^13^C chemical shifts of RIIDD_2_ in the complex were assigned using HC(C)H-TOCSY (62, 63) and ^13^C-edited NOESY-HSQC (59) spectra. Distinct side-chain chemical shifts were observed for Ile3, Gln4, Ile5, and Thr10. Eighty three percentage of sidechain chemical shifts were assigned at least partially (73% completely). The full list of assignments has been deposited in the BioMagResBank database (BMRB accession number 34908).

### Solution structure of the N-MAP2c:RIIDD_2_ complex was determined based on NMR data

**Figure 4:**
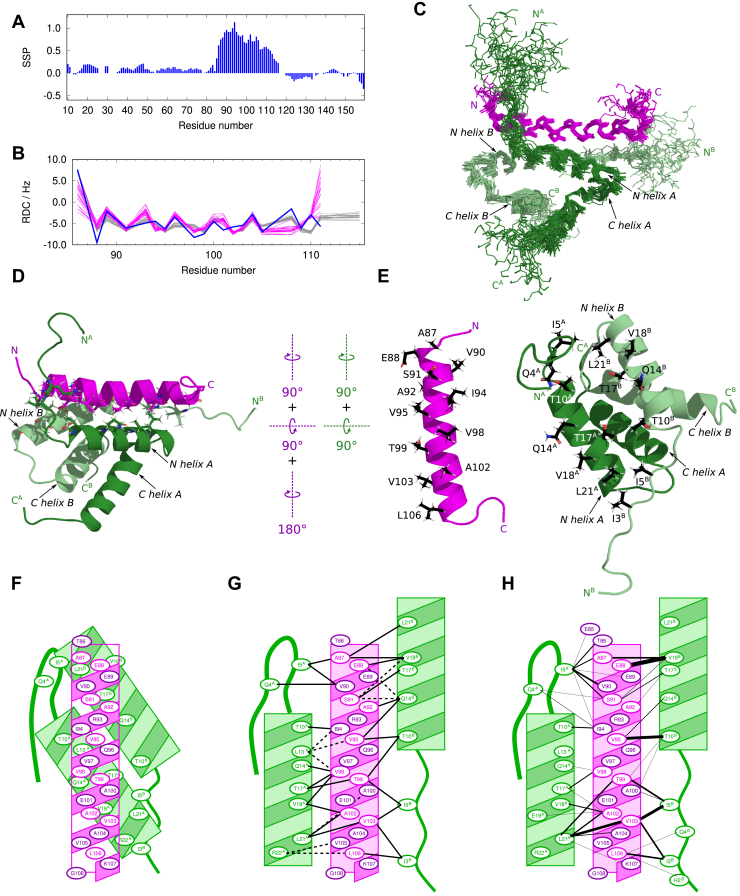
**Solution structure of the N-MAP2c:RIIDD**_**2**_**complex.***A*, SSP of N-MAP2c residues in the complex. *B*, experimental (*blue*) ^1^H-^15^N RDC values of the helical part of N-MAP2c in the complex compared with values calculated from 20 refined structures (*magenta*) and from 25 structures predicted by AlphaFold multimer (*gray*). *C*, backbone traces of a set of 20 superimposed refined N-MAP2c:RIIDD_2_ structures. N-MAP2c, RIIDD_2_ protomers *A* and *B* are shown in *magenta*, *pale green*, and *forest green*, respectively. *D*, a representative refined structure of N-MAP2c:RIIDD_2_ colored as in *panel C*. Side chains of residues involved in the intermolecular contacts are depicted as *sticks*. *E*, the same structure as in panel D rotated by 90° about the vertical and horizontal axes and with the N-MAP2c helix further rotated by 180° to reveal the binding interface. Side chains of amino acids involved in the intermolecular contacts are depicted as *sticks* and labeled with single-letter codes. The N-terminal and C-terminal helices of RIIDD_2_ are labeled as N helix and C helix, respectively. *F*, schematic drawing of the orientation of the N-MAP2c (*magenta*) and RIIDD_2_ (*green*) helices. *G*, contacts between N-MAP2c and RIIDD_2_ helices. *Solid* and *dashed black* lines represent contacts observed in all 20 structures of the refined ensemble and only in some structures of the ensemble, respectively. *H*, intermolecular distance restraints derived from the measured NOE values shown as *black* lines. The width of the line is proportional to the number of restraints per an amino-acid pair (ranging from 1 to 20). Side chains oriented towards the interface are presented in *magenta* and *forest green*; side chains oriented away from the interface are shown in *purple*.

Chemical shifts of assigned residues were used to describe the secondary structure of RIIDD_2_-bound N-MAP2c. The secondary structure propensity calculated from ^1^H^α^, ^13^C^α^, ^13^C^β^, and from backbone amide ^1^H and ^15^N chemical shifts revealed a strong tendency to form an α-helix for Thr86–Gln110 (Fig. 4*A*).

Residual dipolar couplings (RDCs) were measured in the magnetically oriented filamentous phage *Pf1* (64, 65) in order to further probe the structural features of the RIIDD_2_-bound region of MAP2c. In the in-phase, anti-phase spectra (66), peaks of 48 residues were resolved, 20 of which were located in the Thr86–Glu111 region. RDCs of the 18 peaks in the Glu88–Gln110 region correlated well with the values calculated for an α-helix predicted by AlphaFold multimer (67) (version 2.2.0) for residues 87 to 118. On the contrary, RDCs of other residues deviated from the prediction (Fig. 4*B*).

The medium-range NOE between the α-protons of *i*-th and amide protons of (*i* + 3)-th residues, revealing proximity of protons across one turn of the α-helix, were clearly observed for 12 residues with *i* in the range 86 to 105 but missing outside this range. Several protons of Glu109 and Gln110 exhibited NOEs with methyl groups of Ala104 and Val105, respectively. It indicates the formation of a turn between Val105 and Gln110, presumably at Gly108. In summary, combination of the NOE, RDC, and secondary structure propensity data showed that a regular α-helix is formed between Thr86 and Gly108.

The inter-proton distances derived from NOE served as a major source of structural information. The ^15^N- and ^13^C-edited NOESY spectra were complemented with isotope-edited/filtered NOESY spectra, providing specific information about the intermolecular distances.

The NOE cross-peaks were assigned using CYANA (68) in the automated assignment mode. A total of 176 torsion angle restraints predicted by TALOS-N (69) based on chemical shifts, 20 RDC restraints, and α-helical hydrogen bond restraints in regions 13 to 29 and 33 to 49 of RIIDD_2_ and 86 to 110 of MAP2c were also used (77 total). A lower limit was set for the distance between Thr86 and Leu106 of N-MAP2c and for the distance between Ile8 in different RIIDD_2_ subunits to avoid artificial parallel orientation of the RIIDD_2_ protomers. To reduce the computational cost, only residues observed to interact with RIIα in the HNCO experiments (Thr80–Pro120, Fig. 3) were modeled. Out of the 100 structures calculated, a set of 20 structures with the lowest target function was selected. The automated assignment identified a total of 1361 distance restraints based on NOE cross-peaks.

The refinement of the structural model was performed using the CNS software (70, 71) (https://www.ibs.fr/IMG/pdf/sculptorCns_documentation.pdf). First, an unrestrained simulation of the 20 structures with the lowest energy in the CYANA calculation was performed to check the complex stability. While the overall RMSD of the structures was higher, the general fold remained the same. The refinement itself started from an extended structure. A set of 100 structures was calculated using the distance restraints provided by CYANA and the hydrogen bond, torsion angle, and RDC restraints, described above; however the lower distance limit was not used. The 20 lowest energy structures were further refined with an explicit solvent in CNS. As long-range distance restraints were observed only for N-MAP2c residues in the range Glu85–Gln110, only Arg84–Glu111 of MAP2c were included in the final refinement. The obtained set of 20 structures (Fig. 4, *C*–*E*) was deposited in the PDB database under PDB ID 8S8O. Statistics of the restraints and validation are summarized in Table 1.

**Table 1:** Statistics of restraints and validation for structure determination of the N-MAP2c:RIIDD complex

Experimental restraints	
NOE	1316
Torsion angle (TALOS-N)	176
α-helical hydrogen bond	77
RDC	20
NOE restraints per sequence distance	
Intraresidue (|*i*−*j*| = 0)	328
Sequential (|*i*−*j*| = 1)	415
Medium-range (1 < |*i*−*j*| < 5)	310
Intramolecular long-range (|*i*−*j*| ≥ 5)	46
Intermolecular	217
Of which between dimerization domains	78
Of which between RIIDD and MAP2c	139
Calculation statistics	
Rmsd of selected ensemble (ordered parts)	0.88 Å
Energy (average, selected conformers, kcal·mol^−1^)	
Total energy	−4461.12
Bond lengths	57.4956
Bond angles	214.785
Torsion angles	600.358
Van der Waals	−408.571
Electron	−5044.29
NOE restraints	34.4126
Torsion angle restraints	0.023870
RDC restraints	1.67843
Violations (average, selected conformers)	
NOE > 0.5 Å	0
Torsion angle > 5°	0.8
RDC > 2 Hz	0

### N-MAP2c:RIIDD_2_ complex consists of asymmetric four-helix RIIDD_2_ bundle and N-MAP2c helix in a unique orientation

The solved structure consists of the RIIDD_2_ dimer forming the typical antiparallel X-type four-helix bundle and of the amphipathic α-helix of N-MAP2c, interacting with the N-terminal helices of RIIDD_2_ in a similar manner as in the other AKAP:RIIDD_2_ structures (23, 24, 25, 26, 72). The N-MAP2c helix is formed by residues Ala87–Lys107, in agreement with chemical shifts, medium-range distances, and RDC data. Backbone of Thr86 is in the β conformation (*φ ≈* −105°and *ψ ≈ +*155*°*). Both *φ* and *ψ* torsion angles of Gly108 are positive (torsion angles *φ ≈* +90°and *ψ ≈ +55°*), indicating the formation of a turn, which is experimentally supported by Ala104-Glu109 and Val105-Gln110 distance restraints. Orientations of amide NH bonds of residues Thr86–Gln110 agree with the measured RDC values (Fig. 4*B*). The N-terminus of the N-MAP2c helix is confirmed by the RDC value of Thr86, incompatible with the helical conformation. The C-terminus of the helix can be explained by the presence of glycine, a known helix breaker. The experimentally determined extent of the N-MAP2c α-helix differs from the prediction by AlphaFold multimer (67). AlphaFold multimer version 2.2.0 predicts an α-helix for residues Ala87–Asp118 of the N-MAP2c:RIIDD_2_ complex (gray trace in Figs. 4*B* and S3*A* in Supporting Information). Recently released AlphaFold 3 (73) provided a very similar prediction, whereas the experimental data limit the helix to residues Ala87–Lys107. It is interesting that the correct extent of the N-MAP2c α-helix can be found in AlphaFold2 (74) predictions for free MAP2c (cf AF-P15146-F1, AF-Q78DZ1-F1 in UniProt). Note that free MAP2c is known to be disordered and does not exhibit a continuous α-helical propensity for Ala87–Lys107 (6, 34). It documents that structure predictions by AlphaFold2 for free disordered proteins may be biased by the existence of complexes with specific binding partners where certain regions of the studied protein fold.

Binding of the N-MAP2c introduces asymmetry of the N-MAP2c:RIIDD_2_ complex. As shown in Figure 4*G*, different residues of the individual RIIDD_2_ protomers interact with N-MAP2c. The asymmetry of the complex is also reflected by different sets of intermolecular NOEs between N-MAP2c and the individual RIIDD_2_ protomers (Fig. 4*H*). For example, Ile3 of protomer B (Ile3^B^) makes contacts confirmed by multiple NOEs with Val103 and Leu106 of N-MAP2c. These contacts constrain the position of Ile3^B^ in the complex and keep the N-terminus of protomer B in a well-defined extended conformation. For the sake of brevity, we refer to such protomer as “more ordered”. On the other hand, Ile3 of protomer A (Ile3^A^) does not interact with N-MAP2c. Therefore, the conformation of the N-terminus of protomer A is less defined.

**Figure 5:**
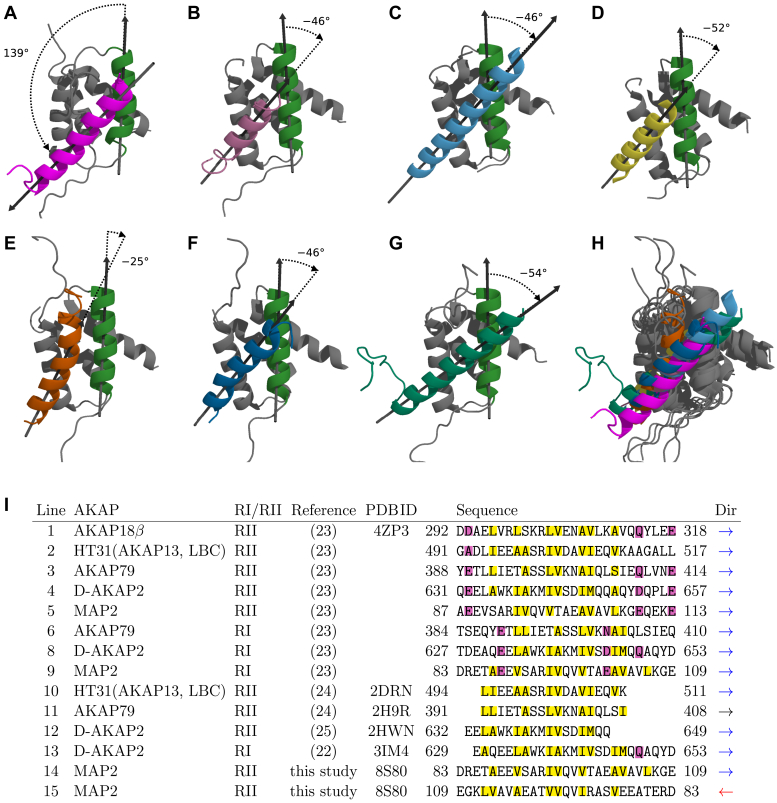
**Aligned structures of AKAP:RIIα-PKAcomplexes.***A*, PDB ID 8S8O (this study), (*B*) PDB ID 2HWN (25), (*C*) PDB ID 4ZP3 (23), (*D*) PDB ID 2IZX (26), (*E*) PDB ID 2DRN (24), (*F*) PDB ID 2H9R (24), (*G*) PDB ID 2KYG (72), (*H*) all structures overlaid. The oriented angles between axes of the more ordered N-terminal helix of RIIDD (shown in *forest green* in all structures) and the AKAP helix are displayed. *I*, alignment of selected AKAP sequences. Alignment with line 1 based on sequence comparison is shown on lines 2 to 9. Polar residues in positions proposed as anchors are highlighted by a *purple* background; hydrophobic residues in positions contacting the RI or RII subunits are highlighted by a *yellow* background. Alignment with line 1 based on the structure comparison is shown on lines 10 to 15. Direction of the sequence is marked by an *arrow* in the last column.

Asymmetry of the AKAP:RII complexes has been thoroughly discussed in the literature. Similar differences in the RII protomer N-termini have been reported for D-AKAP2 by Kinderman *et al.* (25), for AKAP-*IS* (with artificially optimized sequence) by Gold *et al.* (26), and for AKAP18β by Götz *et al.* (23). However, direction of the AKAP helix in these complexes differs from the N-MAP2c:RIIDD_2_ complex: Ile3 in the more ordered N-terminus of the RIIDD_2_ dimer interacts with C-terminal residues of N-MAP2c, but with N-terminal residues of the other AKAPs. In Figure 5, the mutual orientation between N-MAP2c and RIIDD_2_ is measured as the angle between the directions of the AKAP α-helix and of the more ordered N-terminal RIIDD_2_ α-helix. The angle is close to −50° for most AKAP:RIIDD_2_ complexes but approximately *+*140*°* for N-MAP2c:RIIDD_2_. It quantitatively describes that the direction of the N-MAP2c α-helix is reversed and less tilted from the axes of the N-terminal RIIDD_2_ helices, in comparison with other AKAP:RIIDD_2_ complexes.

The interactions between N-MAP2c and RIIDD_2_ are mostly hydrophobic, involving six turns of the N-MAP2c helix. A noteworthy exception is the hydrogen bond between Glu88 and Gln14^B^, observed in some refined structures. The contacts between N-MAP2c and RIIDD_2_ residues are depicted schematically in Figure 4*G* and the interacting residues are displayed in Figure 4*E*. Positions of hydrophobic residues in solved AKAP:RIIDD_2_ structures have been aligned with the MAP2 sequence before we solved the N-MAP2c:RIIDD_2_ structure (23, 24, 26). Newlon *et al.* (24) and Gold *et al.* (26) originally aligned the first hydrophobic contact with the Ala87 of MAP2. Later, Sarma *et al.* (22) observed for D-AKAP2, able to bind both RI and RII subunits of PKA, that the register of the contacts is shifted by one helical turn in the complex with RI compared to the complex with RII (lines 12 and 13 in Fig. 5*I*). Furthermore, Götz *et al.* observed (i) salt bridges between acidic residues of AKAP18β and Arg22 residues of the RIIDD_2_ dimer and (ii) a hydrogen bond between Gln70 of AKAP18β and Gln14 of one RIIDD_2_ helix. Based on these findings, Götz *et al.* proposed that the salt-bridge anchoring determines the specificity of binding to the RI/RII subunits and defines the register and the orientation of the AKAP helix in the complexes (23). The suggested RII-binding motif consists of five hydrophobic patches flanked by three acidic/polar residues. According to this model, the MAP2 residue making the first hydrophobic contact should be Ala87 in complexes with RI (lines 1 and 9 in Fig. 5*I*), but Ser91 in complexes with RII (lines 1 and 5 in Fig. 5*I*). Although the alignment of MAP2 on line 5 of Figure 5*I* perfectly fits the proposed motif, the N-MAP2c helix in our structure interacts with RIIDD_2_ protomer A in a different register, presented on line 14 in Figure 5*I*. This seems to indicate that N-MAP2c actually binds RIIDD_2_ in the register predicted for the RI subunit, corresponding to line 9 in Figure 5*I*. To reconcile the structure with the model proposed by Götz *et al.*, the MAP2 sequence must be aligned in a reversed order with other AKAP sequences (line 15 in Fig. 5*I*). This alignment is given by the interactions of the MAP2c helix with RIIDD_2_ protomer B and reflects the fact that the N-MAP2c α-helix has the opposite orientation in the complex with respect to the more ordered RIIDD_2_ protomer B. The exact orientation of the N-MAP2c helix in the complex with RIIDD_2_ also explains differences in the contacts between N-MAP2c and RIIDD_2_. Glu109 and Glu111 of N-MAP2c are too far to form a salt bridge with Arg22^A^ of RIIDD_2_. Instead, the turn of Gly108 allows for the interaction of Glu109 with Lys107.

In summary, the solved structure of the N-MAP2:RIIDD_2_ complex showed the general similarity with other AKAP:RIIDD_2_ complexes but also revealed features distinct for MAP2. In particular, the orientation of the MAP2 helix differs from AKAPs in other described complexes, resulting in somewhat different intermolecular contacts (39).

### NMR peak broadening revealed MAP2c residues involved in Grb2 binding

**Figure 6:**
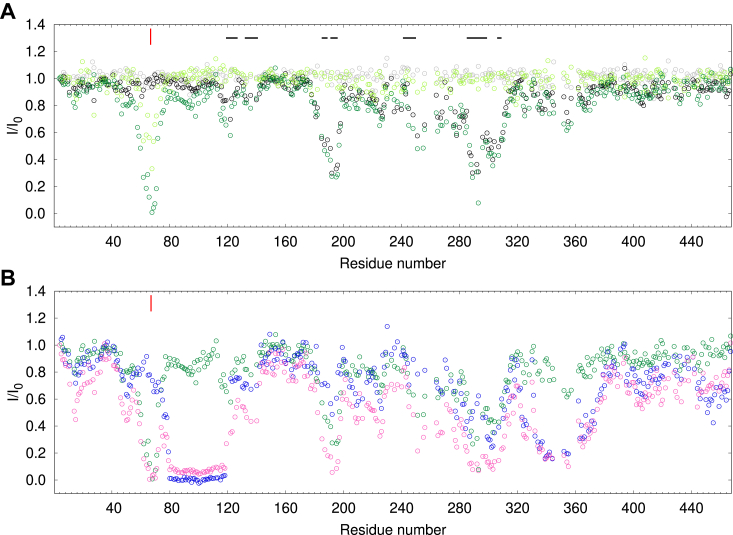
**Interaction of MAP2c with Grb2, Grb2-SH2, and RII*****α*****-PKA monitored by NMR peak broadening.***A*, reduction of peak heights in the HNCO spectra of [^13^C,^15^N]-MAP2c in the presence of equimolar amount of Grb2 and Grb2-SH2. Data for unphosphorylated [^13^C,^15^N]-MAP2c with Grb2 and Grb2-SH2 are shown in *black* and *gray*, respectively; data for Fyn-phosphorylated [^13^C,^15^N]-MAP2c with Grb2 and Grb2-SH2 are shown in *forest* and *pale green*, respectively. *Horizontal* bars above the plots indicate the regions of MAP2c with population of polyproline II conformation higher than 15% for four consecutive residues (6). *B*, reduction of peak heights in the HNCO spectra of [^13^C,^15^N]-MAP2c phosphorylated by Fyn in the presence of equimolar amount of Grb2 (*green*), RIIα-PKA dimer (*blue*), and of both Grb2 and RIIα-PKA dimer (*magenta*). The [^13^C,^15^N]-MAP2c concentration was 100 μM, the degree of Fyn phosphorylation was approximately 75%. The plotted *I*/*I*_0_ values are ratios of the peak heights of the MAP2c complexes to the peak heights of free MAP2c. The *red* marks above the plot indicate the position of pTyr67.

NMR peak broadening was employed to detect MAP2c residues interacting with Grb2 in the same manner as described for the MAP2c interactions with RIIα-PKA. Grb2 can bind MAP2c *via* SH2 or SH3 domains. We have previously identified residues in the variable central region and proline-rich region of MAP2c that interact with the Grb2 SH3 domains (41). In this study, we extended the study of MAP2c interactions to the SH2 domain, which binds MAP2c phosphorylated specifically at Tyr67 (39). We first confirmed a residue-specific phosphorylation of MAP2c on Tyr67 by tyrosine kinase Fyn (Fig. S4 in Supporting Information). Then, we monitored HNCO peak broadening of MAP2c upon interactions with Grb2. Presence of Grb2 reduced peak heights of unphosphorylated MAP2c in several regions (black circles in Fig. 6*A*). The most notable peak broadening was observed for the class I canonical SH3-binding site _288_RTPPKSP_294_. The peak height decreased also in the proline-rich regions (residues 230–300) with multiple minimal SH3-binding PXXP motifs. Even stronger peak broadening was observed in a region between residues 185 and 198, rich in prolines, but lacking the PXXP motif. We compared the peak broadening with our previous conformational analysis (6). The comparison showed that SH3 domains of Grb2 interact with regions of increased population of polyproline II conformation (horizontal bars in Fig. 6*A*).

Additional peak broadening of Fyn-phosphorylated MAP2c (compared to unphosphorylated MAP2c) in the presence of Grb2 revealed a phosphorylation-dependent binding site around pTyr67. The peak height was reduced below 50% for residues 60 to 70 (forest green circles in Fig. 6*A*). Broadening of the peak of pTyr67 below the detection limit showed that pTyr67 became a part of a large, well-ordered complex in the presence of Grb2. The same experiment was performed with Fyn-phosphorylated MAP2c and Grb2-SH2. Peak broadening was observed in the same region of the MAP2c sequence as with full-length Grb2 (pale green circles in Fig. 6*A*), confirming that pTyr67 interacts with the SH2 domain. The less deep drop of the peak height may be a combination of two factors. First, the size of the complex with Grb2-SH2 does not exceed the limit of conventional NMR experiments and thus the peak height of residues in the complex is not negligible. Second, Grb2-SH2 is not fully saturated by Fyn-phosphorylated MAP2c at the concentrations used in the experiment (see below).

In summary, phosphorylation at Tyr67 of rat MAP2c introduces a new binding site, interacting with the SH2 domain of Grb2. In contrast to the RIIα-PKA binding, the interaction with Grb2-SH2 involves only few residues in the vicinity of pTyr67.

### Chemical shift perturbation identified the binding interface of the MAP2c:Grb2-SH2 complex

We characterized the interaction interface of the complex of MAP2c with Grb2-SH2 using the chemical shift perturbation (CSP). The method is based on the fact that chemical shifts are perturbed by changing the electron distribution in the proximity of the observed atomic nucleus, *for example*, by ligand binding. Whereas exact interpretation of the chemical shift changes is difficult, a quick semiquantitative analysis can be performed. We assume that the effect of the binding decreases with the distance of the observed nucleus from the binding site. Furthermore, we can distinguish a fast equilibrium between the free and bound protein, typical for weak affinity, from a slow exchange between free protein and usually tightly bound complex. In both cases, dissociation constants can be estimated if the proper concentration range is covered in a titration series. In the former case, observed in our study, *K*_D_ is calculated from gradual chemical shift changes of a single peak moving in the spectrum during the titration. The concentration ratio of the free and bound form is assumed to correspond to the chemical shift change relative to the difference between the chemical shifts of free and completely bound protein. If double-resonance or triple-resonance spectra are analyzed, it is advantageous to combine chemical shift changes of individual correlated nuclei into one value called combined chemical shift perturbation (CCSP).

**Figure 7:**
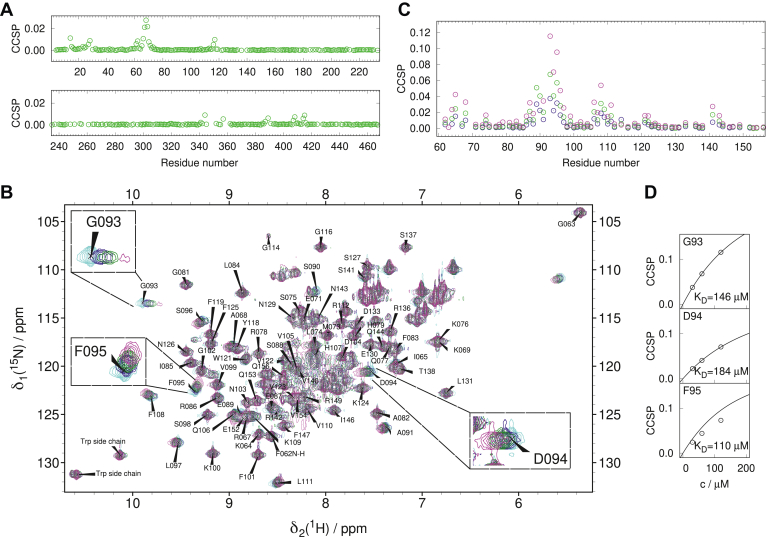
**Interaction of Grb2-SH2 with MAP2c monitored by chemical shift perturbation.***A*, CCSP of 100 μM Fyn-phosphorylated [^13^C,^15^N]-MAP2c upon addition of 100 μM [^15^N]-Grb2-SH2. *B*, 2D ^1^H,^15^N HSQC spectra of 80 μM [^15^N]-Grb2-SH2 recorded during titration with unlabeled Fyn-phosphorylated MAP2c. Peaks exhibiting the largest CCSP are shown in insets. *C*, CCSP of 80 μM [^15^N]-Grb2-SH2 during the titration with unlabeled Fyn-phosphorylated MAP2c. *D*, fits of CCSP values of Gly93, Asp94, and Phe95 of Grb2-SH2 providing estimation of the dissociation constant of Grb2-SH2 in complex with Fyn-phosphorylated MAP2c. *Cyan*, *blue*, *green*, and *magenta* colors in *panels B* and *C* correspond to 0 μM, 40 μM, 80 μM, and 160 μM MAP2c concentrations, respectively. The degree of Fyn phosphorylation was approximately 75%.

To map the binding site of MAP2c, we recorded 3D HNCO spectra of 100 μM [^13^C,^15^N]-MAP2c phosphorylated to 75% by Fyn at Tyr67 with and without 100 μM unlabeled Grb2-SH2 and evaluated CSP. CCSP of ^1^H, ^13^C, and ^15^N is presented in Figure 7*A*. The obtained profile agreed with the results of the peak broadening. The most distinct CCSP was observed for pTyr67 and several neighboring residues upon binding to Grb2-SH2. Less distinct CCSP with smaller number of residues involved were observed in some other regions, namely in the very N-terminal part of MAP2c and within the microtubule-binding domain. These small CCSP values most likely reflect that MAP2c forms transient compact structures (6). Residues distant in the sequence thus may temporarily get close to pTyr67 and be influenced by interactions with Grb2-SH2.

In a complementary experiment, we acquired 2D ^1^H,^15^N HSQC spectra of 80 μM [^15^N]-Grb2-SH2 with 40 μM, 80 μM, and 160 μM unlabeled MAP2c phosphorylated to 75% by Fyn at Tyr67 (Fig. 7*B*). Binding of MAP2c phosphorylated by Fyn affected chemical shifts mainly in the region of Grb2-SH2 comprising residues Arg86–Leu97 and to a lesser extent Lys64–Ala68 and Gln106–Leu111. Small changes in the chemical shift were observed for Trp121, Val122, and for few residues close to the C terminus. To verify that the phosphorylation at Tyr67 is responsible for the Grb2-SH2 binding, the same experiments with unphosphorylated MAP2c were performed (Fig. S5, *C* and *D* in Supporting Information). No significant CCSP was observed in MAP2c or in the SH2 domain.

The peaks influenced by the presence of Fyn-phosphorylated MAP2c moved in the spectra of [^15^N]-Grb2-SH2 in a linear manner typical for the two-state binding model of fast exchange between free and bound form. The calculated CCSP values are plotted in Figure 7*C*. The dissociation constant of the complex estimated from CCSP of Gly93, Asp94, and Phe95 was (147 ± 37) μM (Fig. 7*D*). The relatively high value is consistent with the observed fast exchange. The high-micromolar affinity may seem too weak to be physiologically relevant. However, MAP2c interacts also with the SH3 domains of Grb2 (37, 41) and the effective local concentration of the SH2 domain is increased by prior binding to the SH3 domains.

Our next goal was to structurally characterize the complex of Grb2-SH2 with Fyn-phosphorylated rat MAP2c. When structures of complexes are solved by NMR in the manner described above for N-MAP2c:RIIDD_2_, it is desirable to prepare samples containing the isotope-labeled component almost completely in the bound form. The estimated value of the dissociation constant of Grb2-SH2 in complex with Fyn-phosphorylated MAP2c showed that an almost complete formation of the complex would require higher concentrations of the proteins than could be achieved using our protocols. Therefore, we did not attempt to solve the solution structure of the complex with Grb2-SH2 using distance restraints, as we did for the N-MAP2c:RIIDD_2_ complex. Instead, we used CCSP to map the binding site of Grb2-SH2 in complex with Fyn-phosphorylated MAP2c.

**Figure 8:**
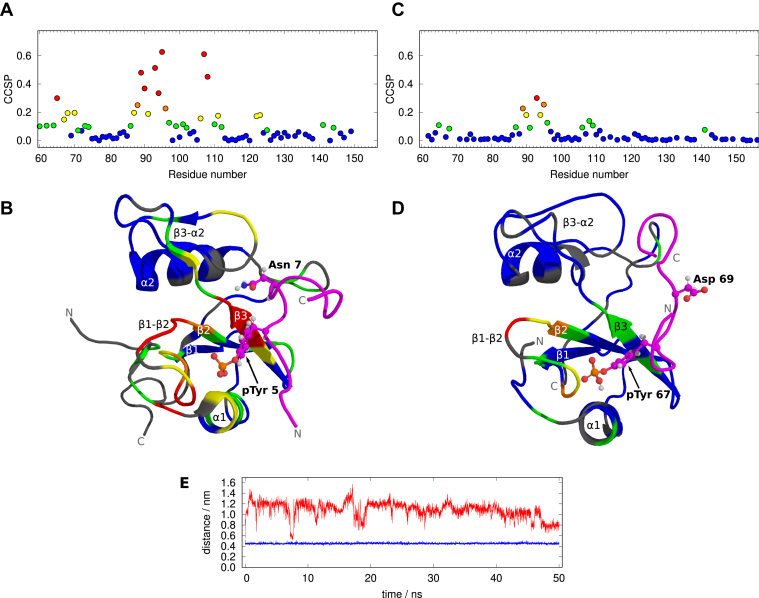
**Comparison of Grb2-SH2 in complexes with phosphoTyr-Val-Asn adn with MAP2c phosphorylated at Tyr67.***A*, CCSP of Grb2-SH2 in complex with phosphoTyr-Val-Asn (75). *B*, structure of Grb2-SH2 in complex with a phosphopeptide containing a pYVN motif (PDB ID 1QG1) (76), colored according to the CCSP plotted in *panel A*. Grb2-SH2 residues with missing CCSP values are shown in *gray*, the backbone trace of the phosphopeptide is shown in *magenta*. *C*, CCSP of Grb2-SH2 bound to rat MAP2c. The displayed CCSP values are calculated from the estimated *K*_D_ for Grb2-SH2 fully saturated by Fyn-phosphorylated MAP2c. *D*, the lowest-energy structure of the molecular dynamics trajectory simulated for Grb2-SH2 in complex with Fyn-phosphorylated MAP2c, colored according to the CCSP plotted in *panel C*. Grb2-SH2 residues with missing CCSP values are shown in *gray*, the backbone trace of Fyn-phosphorylated MAP2c is shown in *magenta*. Residues pTyr5 and Asn7 of the pYVN motif and pTyr67 and Asp69 of Fyn-phosphorylated MAP2c are displayed as sticks and balls in *panels B* and *D*. *E*, distances between C^γ^ of the phosphopeptide Asn7/MAP2c Asp69 and carbonyl carbon of Grb2-SH2 Lys109 monitored during molecular dynamics simulation of Grb2-SH2 with phosphopeptides containing the pYVN sequence (*blue*) and the sequence derived from Fyn-phosphorylated MAP2c (*red*).

The elevated CCSP values identify the binding interface of the studied complex. Similar data were reported for Grb2-SH2 in the complexes of known structures. We used a complex of Grb2-SH2 with tightly bound phosphoTyr-Val-Asn (pYVN) peptide (75) as a reference. Binding of pYVN (and other peptides sharing the pYXN motif) to Grb2-SH2 results in large CCSP in loop β1-β2 and strand β3 of the SH2 domain (Fig. 8*A*). Smaller CCSP are observed in the N-terminal region of helix α1 and in the C-terminal half of the long loop β3-α2. Structure of Grb2-SH2 (76) colored according to the CCSP upon pYVN binding (75) is presented in Figure 8*B*. The large CCSP shifts are caused by burying pTyr to a pocket formed by the β-sheet, helix α1, and loop β1-β2, and by burying phosphopeptide Asn into the hydrophobic pocket between strand β3 and loop β3-α2, where it forms a hydrogen bond with backbone carbonyl oxygen of Lys109.

To provide a direct comparison, we have extrapolated CCSP of Grb2-SH2 obtained in our study to the value corresponding to the full saturation of Grb2-SH2 by Fyn-phosphorylated MAP2c (Fig. 8*C*). Relatively large CCSP was observed in loop β1-β2 but much weaker CSP in strand β3 (note that strand β3 is also influenced by pTyr). Moreover, the moderate CSP of Trp121 and Val122 in loop β3-α2 of pYVN is negligible in the complex with rat MAP2c. It confirms that positions of human Asn69 and rat Asp69 differ in the complexes, which explains different affinities of the pYXN and pYXD motifs (77). We speculate that Asp69 of rat MAP2c, which is charged and unable to form the hydrogen bond with carbonyl of Lys109, does not enter the hydrophobic pocket like Asn69 in the pYXN motif and thus does not influence strand β3. To test this hypothesis and to visualize the Grb2-SH2:Fyn-phosphorylated MAP2c complex, we performed molecular dynamics simulation of Grb2-SH2 with phosphopeptides containing Asn and Asp in the position +2 from pTyr.

The simulations started from the 1QG1 structure (76), where the side-chain amide of Asn7 in the pYVN motif is buried between strand β3 and loop β3-α2 of Grb2-SH2 and is hydrogen-bonded to backbone carbonyl oxygen of Lys109 of Grb2-SH2, as mentioned above. The pYXD motif was created by *in silico* mutation of the sequence Val6-Asn7-Val8 of the bound phosphopeptide to Ser6-Asp7-Thr8, followed by equilibration. Overlayed snapshots of phosphopeptide conformations during both simulations are presented in Fig. S6, *A* and *B* in Supporting Information. We monitored the distance between C^γ^ of the phosphopeptide Asn7/Asp7 and carbonyl carbon of Lys109 (Fig. 8*E*). The distance remained close to 0.45 nm for Asn during the whole simulation, confirming presence of the hydrogen bond with Lys109. However, the distance fluctuated between 0.7 nm and 1.6 nm for pYXD, documenting that the aspartate is not buried between strand β3 and loop β3-α2 and does not interact with the carbonyl of Lys109. The distance between phosphate oxygen of pTyr and N^η1^ of Arg86 remained close to 0.2 nm for both phosphopeptides, confirming that both complexes remained stable during the simulation (Fig. S6, *C* and *D* in Supporting Information). The simulation thus supported our hypothesis, as the aspartate was not inserted between strand β3 and β3-α2 loop and did not interact with the carbonyl of Lys109. We can therefore use the simulated structure with the lowest energy as a model of Grb2-SH2 bound to the Fyn-phosphorylated MAP2c and compare it to the experimental CCSP data.

Figure 8*D* shows our structural model colored according to the extrapolated CCSP values obtained in our study. The colors document that the chemical shifts in loop β1-β2 are greatly influenced by buried pTyr67, but the chemical shifts in strand β3, distant from Asp69 of Fyn-phosphorylated MAP2c, are perturbed much less.

In summary, we used chemical shift changes to map the interaction interface between Grb2-SH2 and Fyn-phosphorylated MAP2c. Combination of molecular dynamics simulations with the analysis of experimental CCSP values explained structurally how binding of Grb2-SH2 to rat Fyn-phosphorylated MAP2c differs from the interactions of Grb2-SH2 with phosphopeptides of the pYXN sequence.

### RIIα-PKA and Grb2 bind to MAP2c simultaneously

The major binding sites recognized by RIIα-PKA and Grb2 in the N-terminal region of MAP2c are located close to each other. Therefore, we also investigated whether MAP2c phosphorylated by Fyn at Tyr67 is able to interact with both the binding partners simultaneously or whether interaction with one of the proteins prevents binding of the other one. NMR peak broadening was employed in the same manner as described for the individual interactions of RIIα-PKA and Grb2 with MAP2c (Fig. 6*B*). Resolution of the 3D HNCO NMR spectra allowed us to monitor the possible interference of RIIα-PKA and Grb2 binding for individual residues. The drop of the peak height in the presence of both RIIα-PKA and Grb2 (magenta circles) was very close to the combination of the effects of the individual proteins (RIIα-PKA shown in blue or Grb2 shown in green). In particular, the drop of the peak height in the vicinity of phospho-Tyr67 due to binding to Grb2 was not influenced at all by RIIα-PKA in a concentration at least five orders of magnitude higher than *K*_D_ of the MAP2c:RIIα-PKA complex. Similarly, the drop of the peak heights of residues Thr80–Asp118 due to RIIα-PKA binding was not influenced by the presence of 100 μM Grb2. The small differences between residual peak heights are due to a slight deviation of the concentrations from the equimolar ratio (the ∼8% deviation is within the error of concentration measurement). The independence of binding was observed also outside the major interaction sites in the N-terminal domain of MAP2c. For example, the drop of peak heights of MAP2c residues Gln332–His344, binding RIIα-PKA but not Grb2, is not influenced by the presence of Grb2. In overlapping binding sites (*e.g.* residues 180–200, 280–310), the effects of RIIα-PKA and Grb2 binding cumulate the following: the peak heights shown in magenta (presence of both RIIα-PKA and Grb2) are lower than those shown in green (presence of Grb2) and blue (presence of RIIα-PKA). The lack of interference at 100 μM concentrations of the binding partners suggests that RIIα-PKA and Grb2 are able to bind MAP2c phosphorylated at Tyr67 simultaneously at physiological conditions. We also tested the possible competition between RIIα-PKA and Grb2 quantitatively by ITC. Fyn-phosphorylated MAP2c was titrated by RIIα-PKA in the absence and presence of 100 μM Grb2. No obvious effect of Grb2 on the RIIα-PKA binding was observed (Fig. S7 in Supporting Information).

In conclusion, we probed a possible interference of interactions of Fyn-phosphorylated MAP2c with RIIα-PKA and Grb2 in a residue-specific manner. The results showed that RIIα-PKA and Grb2 are able to bind MAP2c simultaneously, in spite of the large size of the proteins and of the close proximity of their binding sites on MAP2c.

## Discussion

We have investigated the interactions of two binding sites in the N-terminal domain of MAP2c that distinguish MAP2 from Tau. The interactions with RIIα-PKA and Grb2 interfere with different signaling pathways and also differ in structural features and affinities. Our results suggest that RIIα-PKA and Grb2 can interact with MAP2c simultaneously, without an obvious mutual interference.

The first interaction characterized in this study was the binding of MAP2c to the regulatory subunit RIIα of PKA. ITC revealed that MAP2c and N-MAP2c bind RIIα-PKA and RIIDD_2_ with nanomolar affinity. NMR peak broadening of MAP2c residues Thr80–Asp118 showed that these amino acids did not exhibit features of disordered protein in the presence of RIIα-PKA or RIIDD_2_. The solution structure of N-MAP2c in complex with RIIDD_2_ was solved by NMR (Fig. 4). Although the structures of several AKAP:RIIDD_2_ complexes have been determined previously (23, 24, 25, 26, 72), our results answered three questions that could not be resolved without a direct experimental evidence: what is the exact length, register, and orientation of the amphipathic helix of N-MAP2c, predicted to interact with RIIDD_2_. The solved structure revealed that the orientation of the MAP2 helix interacting with RIIDD_2_ differs from the known AKAP:RII complexes. Such structural information not only fills a gap in our knowledge but is important for following studies. For example, electron-microscopic studies contribute to better understanding of the role of AKAP anchoring in the regulation of PKA activity. Such studies rely on high-resolution structures of well-defined structural units of the studied large and dynamic molecular assemblies (78). One of such structural units is the determined structure of the N-MAP2c:RIIDD_2_ complex. In addition to the directly biologically relevant results, solving the N-MAP2c:RIIDD_2_ structure allowed us to test the current performance of sequence-based predictors (AlphaFold multimer, AlphaFold 3) of protein structures (67, 73, 74). Results of the test showed that predictions of complexes including disordered proteins folded upon binding still need experimental validation. This is useful information not only for structural biologists but also for a broader community of users of such computational tools.

The second investigated interaction was the binding of MAP2c phosphorylated at Tyr67 to Grb2-SH2. We have found that rat MAP2c phosphorylated by Fyn kinase binds Grb2-SH2 with a relatively low affinity. The CSP analysis supported by molecular dynamics simulations provided an insight into structural differences between complexes of Grb2-SH2 with phosphopeptides containing the pYXN and pYXD sequence motifs. The results showed that phosphopeptides with the pYXD sequence can bind Grb2-SH2. However, replacement of Asn in the position +2 by Asp leads to the loss of an important interaction with Grb2-SH2 Lys109. It substantially reduces the binding affinity. Knowledge of impact of these motifs on the binding interface is important because the difference between Asn and Asp in the position +2 from pTyr distinguishes not only different SH2-binding proteins but also MAP2 in different organisms (see below).

The variability in the residue at position 69 (using numbering of the rat protein) with strong structural and functional consequences inspired us to compare also other motifs in the MAP2 sequence associated with Grb2 and RIIα-PKA binding. We have identified regions of MAP2c interacting with the Grb2 SH3 domains in our previous study (41). In addition to the class I SH3-binding motif _288_RTPPKSP_294_, Grb2 interacts with residues 185 to 198 in the variable central region of MAP2c (*cf.* Fig. 6). The MAP2c sequence between Pro179 and Pro194 contains five prolines and tends to form polyproline II conformation (6) but does not contain any SH3-binding PXXP motif. Both binding sites have identical sequences in human and rat MAP2c. The region of the sequence including the Grb2-SH2 and RIIα-PKA binding sites is also very similar in human and rat MAP2c. The only difference between residues 55 and 119 is Asn69 in human MAP2c *versus* Asp69 in rat MAP2c. Remarkably, Asn69 is the residue-determining specificity of the interaction with Grb2. Asparagine at the position +2 from the phosphorylated tyrosine is also present in receptor tyrosine kinases and other proteins interacting with the SH2 domain of Grb2 (42, 79, 80). This is in a good agreement with the relatively low affinity of Fyn-phosphorylated rat MAP2c to Grb2-SH2 observed in our study. Whereas the typical dissociation constants of complexes of Grb2-SH2 with peptides containing the pYXN motif are low-micromolar range (81, 82, 83), we obtained a value at least an order of magnitude higher.

The physiologically important interaction sites have usually conserved amino acid composition. This is also true for most binding partners of the Grb2-SH2 (see alignment of the Grb2 SH2-binding sequences of EGFR and CD28 in Supporting Information, Table S1). In the case of the Grb2-SH2 binding site of MAP2, we observe the opposite. Residue 69 of MAP2, determining the specificity for Grb2-SH2 binding, is one of least conserved amino acids in the discussed region among mammals. Comparison of MAP2 sequences of several mammalian species in Table 2 shows that the residue 69 can be Asn or Asp, but Arg is found in Prototheria (*e.g.* platypus *Ornithorhynchus anatinus*) and Marsupialia (*e.g.* koala *Phascolarctos cinereus*). The variations between Asn and Asp are observed even within the same order of placentals. In other vertebrates, residue 69 is even more variable. Moreover, Tyr67 is not conserved in amphibians and the whole motif is often missing in fish (Actinopterygii). It opens a question of the biological relevance of this variability.

**Table 2:** Alignment of selected vertebrate sequences corresponding to the human Grb2-SH2 binding motif pYXN

Class	Order	Binomial name	Sequence
Mammalia	Primates	*Homo sapiens*	HGSQGTYS**N**TKENGI
Mammalia	Primates	*Macaca fascicularis*	HGSQGTYS**N**TKENGI
Mammalia	Rodentia	*Rattus norvegicus*	HGSQGTYS*D*TKENGI
Mammalia	Lagomorpha	*Lepus europaeus*	HGSQATYS**N**TKENGI
Mammalia	Chiroptera	*Pteropus giganteus*	HGSQSTYS*D*TKENGI
Mammalia	Chiroptera	*Myotis myotis*	HGSQSTYS**N**TKENGI
Mammalia	Carnivora	*Lynx canadensis*	HGSQSTYS*D*TKENGI
Mammalia	Artiodactyla	*Sus scrofa*	HGSQGTYS*D*TKENGI
Mammalia	Artiodactyla	*Cervus canadensis*	HGSQGTYS**N**TKENGI
Mammalia	Diprotodontia	*Phascolarctos cinereus*	HGQQGTYSRTKENGI
Mammalia	Monotremata	*Ornithorhynchus anatinus*	REQQTTYPRAKENGI
Aves	Passeriformes	*Serinus canaria*	HEQPGTYAQTKENGI
Aves	Galliformes	*Phasianus colchicus*	REQPGTYAHSKENGI
Aves	Falconiformes	*Falco rusticolus*	HEQPGTYARTKENGI
Aves	Columbiformes	*Columba livia*	HEQPGTYACTKENGI
Aves	Casuariiformes	*Dromaius novaehollandiae*	QEQQGTYSRTKENGI
Reptilia	Squamata	*Anolis carolinensis*	RGQQSSYSRTKENGI
Reptilia	Testudines	*Caretta caretta*	RGQQGTYSRTKENGI
Amphibia	Gymnophiona	*Geotrypetes seraphini*	HGHQASYHRTKENGI
Amphibia	Anura	*Bufo bufo*	RDLQESHATSKENGI
Actinopterygii	Polypteriformes	*Erpetoichthys calabaricus*	HGP-DSYAAAKENGF
Actinopterygii	Lepisosteiformes	*Lepisosteus oculatus*	ESHAAPYSAARENGF
Actinopterygii	Cypriniformes	*Danio rerio*	QPGAGSATYAKENGF

Tyrosines corresponding to human Tyr67 are underlined; asparagines and aspartates aligned with human Asn69 are shown in *bold* and *italics*, respectively.

The explanation that replacement of human Asn69 by Asp69 in rat reflects a difference in the SH2 domain can be excluded, as human and rat Grb2 have identical sequences. Another possibility is that the interaction of pTyr67 with Grb2-SH2 is an accidental fact or an evolutionary relic without any biological function. This hypothesis is supported by a relatively weak evidence of a direct physiological role of pTyr67. Interactions of MAP2 with Grb2 have been confirmed (38, 50) but without showing that binding of pTyr67 to the SH2 domain is important *in vivo*. Perhaps the strongest evidence of the importance of pTyr67 was provided by Zamora-Leon *et al.* (84), who showed that transfection of COS7 cells with Fyn and MAP2c increased process outgrowth in an additive manner. Mutation of Tyr67 or Tyr50 to phenylalanine influenced the length of the processes but did not prevent the initiation of process outgrowth. The observed effect of the Y50F mutation seems to contradict the essential role of Tyr67. The authors concluded that the effect of the Y50F mutation was caused by altered MAP2c conformation. However, it cannot be excluded that factors other than pTyr67 contributed to the outgrowth stimulation.

Finally, different affinities of pTyr67 of MAP2 for Grb2-SH2 in different species may represent a fine tuning of the control of coupling receptor kinases to the Ras signaling pathway by Grb2 (at least in mammals). Complete binding of MAP2 to Grb2 would require higher phosphorylation levels for Asp (or another residue) than for Asn at the position 69. Therefore, the response (in terms of MAP2 competing with the RTK binding to Grb2) to the phosphorylation at Tyr67 differs in species with different residue at the position 69. Finding which of the discussed hypothesis is correct would require further studies.

## Experimental procedures

### DNA constructs

The plasmid coding for WT rat MAP2c (UniProt ID A6KFC7, plasmid pET3d-MAP2c) was a kind gift from Pr. Wiche (University of Vienna). The plasmids coding for human RIIα-PKA (UniProt ID P13861, plasmid pET28-RIIα-PKA) was a kind gift from Enno Klussmann (Max Delbrück Centrum für Molekulare Medizin). DNA encoding the dimerization/docking domain of RIIα-PKA (RIIDD_2_) was amplified by PCR using the primers 5′-atcatgccatgggcatgagccacatccagatccc and 5′-attcgcggatccttatgaggctggggcgcggg and the pET28-RIIα-PKA plasmid as template. The PCR product was cloned in pETM11 by restriction cutting using NcoI and BamHI and ligation. N-MAP2c was cloned in the pETM11 vector and MAP2c 300 to 467 was cloned in pET28 vector, as described previously (41). The MAP2c fragment 159 to 467 was amplified by PCR using the primers 5′-atcatgccatggctgctcccagtgcgtttaaacagg and 5′-attcgcggatccttacaagccctgcttagcgagcg and pET3d-MAP2c as the template. The PCR product was cloned in pETM11 by restriction cutting using NcoI and BamHI. The plasmid coding for His-tagged WT human Grb2 (UniProt ID P62993, plasmid pET28-His-Grb2; NP 002077.1) was a kind gift by JCD Houtman (85). Two mutations (C32S, C198A) were introduced based on the work of Yuzawa *et al.* (86) to improve the stability of the protein as described previously (41). The SH2 domain of human Grb2 (residues 57–158) was cloned into the pET28 plasmid with an N-terminal His-Tag, followed by a thrombin protease cleavage site (MGSSHHHHHHSSGLVPRGSHM; pET28-His(Thr)-hGrb2 SH2 (residues 57–158)), and the integrity of the generated vector confirmed by sequencing. pET21-muABL1 (138–534, F420V)-His, a segment of the murine Abl gene (UniProt ID P00520-1), covering the SH2 domain and the kinase domain, was amplified from a pGEX-Abl construct (aa 139–543, F420V) kindly provided by Dr Jean Wang (87). With the amplification primers (CGGGCTAGCGTCAACAGCCTGGAGAAAC and GCGCTCGAGCGTGCCTCGTTTC), restriction sites for NheI and XhoI were introduced at the ends. The plasmid coding for *Yersinia* tyrosine phosphatase YopH (pCDFDuet-YopH(164–468)) was obtained from Addgene (88) (plasmid # 79749; http://n2t.net/addgene:79749).

### Protein expression and purification

Expression and purification of MAP2c, MAP2c with a hexahistidine tag MKHHHHHHPMSDYDIPTTENLYFQGA at its N terminus (H6-MAP2c), MAP2c fragments 159 to 467 and 300 to 467, and RIIα-PKA was described earlier (41, 89), using 4 mM 2-mercaptoethanol during purification. [^13^C,^15^N]-N-MAP2c and [^13^C,^15^N]-RIIDD_2_ were expressed in M9 medium containing 2g·l^−1^ [^13^C]-glucose and 1g·l^−1 15^NH_4_Cl. The N-terminal MAP2c fragment including residues 1 to 159 and the hexahistidine tag at its N terminus (N-MAP2c) was expressed in *Escherichia coli* BL21(DE3)RIPL overnight at 20 °C. N-MAP2c was purified on HisTrap column (GE Healthcare) chelated with Ni^2+^ions in 20 mM Tris, 500 mM NaCl, 20 mM imidazole and eluted with a 0 to 500 mM imidazole gradient. After dialysis into 20 mM Tris, 50 mM NaCl, pH 7.5, the protein was further purified on HiTrap Q column (GE Healthcare) in 20 mM Tris, pH 8 and eluted with a gradient of 0 to 500 mM NaCl. The [^13^C,^15^N]- RIIDD_2_ was expressed in *E. coli* BL21(DE3)RIPL overnight at 20 °C. The protein was purified on HisTrap HP column (Cytiva) in 20 mM Tris, 500 mM NaCl, 20 mM imidazole and eluted with a 0 to 500 mM imidazole gradient and dialyzed into 50 mM Tris, 100 mM NaCl, pH 7.5. The hexahistidine tag was cleaved with TEV protease overnight at room temperature. The cleaved tag was removed using HisTrap HP column and RIIDD_2_ was collected as flowthrough. The protein was diluted 6 × in 20 mM Tris, pH 8.0 to reduce NaCl concentration and purified on HiTrap Q HP (Cytiva) column in 20 mM Tris, pH 8.0 and eluted with a gradient of 1 M NaCl. Unlabeled RIIDD_2_ was expressed in 2 × YT medium and purified following the same protocol. All proteins were dialyzed in NMR buffer (50 mM MOPS, 100 mM NaCl, pH 6.9) overnight.

N-MAPc labeled with [^13^C,^15^N]-Ile,Val, and [^15^N]-Leu was expressed in 2 l of M9 medium with 50 μg ·ml^−1^ kanamycin and left to grow at 37 °C until A600 reached the value 1.0. 5 g ·l^−1^ unlabeled Glu, 0.1 g ·l^−1^ unlabeled Ala, Arg, Asp, Phe, Cys, Gly, 0.1 g ·l^−1^ [^13^C,^15^N]-Ile, 0.1 g ·l^−1^ [^13^C,^15^N]-Val, and 0.1 g ·l^−1^ [^15^N]-Leu were added to the culture and the expression was induced after 15 min with 0.4 mM IPTG. The protein was purified as described for [^13^C,^15^N]-N-MAP2c. For the assignment, the hexahistidine tag was cleaved with TEV protease overnight at room temperature, the tag was removed, and the untagged protein was purified as described above.

MAP2c labeled with [^15^N]-Tyr was expressed in *E. coli* BL21(DE3)RIPL. The bacterial culture was grown in 800 ml of M9 medium (pH 7.4) supplied with 2 g of glucose, 1 g of NH_4_Cl, and 100 mg of each of the 20 standard proteinogenic unlabeled amino acids except tyrosine at 37 °C until A600 reached the value 1.0. One gram of each of the 20 standard proteinogenic unlabeled amino acids dissolved in 200 ml of H_2_O except tyrosine and 100 mg of [^15^N]-Tyr dissolved in 10 ml of H_2_O were added to the culture. The expression was induced after 15 min with 0.4 mM IPTG and the culture was pelleted after 4 h (the protocol was taken from (90) and modified for our needs). The protein was purified as described earlier for unlabeled and [^13^C,^15^N] MAP2c (41).

Grb2 was expressed and purified as described earlier (41). ^15^N-labeled SH2-Grb2 was expressed in *E. coli* BL21(DE3)RIPL in M9 medium containing 1g·l^−1 15^NH_4_Cl and 2 g ·l^−1^ unlabeled glucose overnight at 20 °C. The pellet was resuspended in 50 mM Tris, 300 mM NaCl, 20 mM imidazole, 1 mM PMSF, pH 8.0 and sonicated. The protein was purified on HisTrap HP column (Cytiva) chelated with Ni^2+^ions using 50 mM Tris, 200 mM NaCl, 500 mM imidazole, pH 8.0 for elution (0–100% gradient). After dialysis to 50 mM Tris, 200 mM NaCl, 1 mM EDTA, pH 8.0, SH2-Grb2 was purified with Superdex S75, concentrated, and dialyzed to NMR buffer. We decided not to cleave off the hexahistidine tag because we observed that the presence of the tag helps to keep the Grb2 SH2 domain in its monomeric form.

c-Abl was expressed in *E. coli* BL21(DE3) containing pCDFDuet-YOPH in TB medium at 18 °C for 16 h at 90 rpm. The pellet was resuspended in 50 mM Tris, 500 mM NaCl, 5% glycerol, 25 mM imidazole, lysozyme (1 mg/ml), 0.1 mM PMSF, pH 8.0. The protein was purified on HisTrap HP column (Cytiva) chelated with Ni^2+^ions using 50 mM Tris, 500 mM NaCl, 5% glycerol, 250 mM imidazole, pH 8.0 for elution (0–50% gradient) and dialyzed to 50 mM Tris, 100 mM NaCl, 5% glycerol, 5 mM DTT, pH 8.0.

### Isothermal titration calorimetry

For ITC experiments, MAP2c, N-MAP2c, MAP2c 159 to 467, MAP2c phosphorylated by Fyn, RIIα-PKA, RIIDD_2_, and Grb2 were dialyzed overnight in 50 mM MOPS, 100 mM NaCl, 4 mM β-mercaptoethanol. Protein concentration was determined by NMR (91) using 1 mM ubiquitin (Asla biotech) as a reference sample and verified by amino-acid analysis (MAP2c, N-MAP2c, MAP2c 159–467, MAP2c-Fyn, and Grb2) or using the Bradford method (RIIα-PKA and RIIDD_2_). Binding of MAP2c or N-MAP2c to RIIα-PKA was carried out at 27 °C using Auto PEAQ-ITC (Malvern Instruments). One microliter volumes of 517 μM or 600 μM RIIα-PKA or RIIDD_2_ were injected with a microsyringe into the 340 μl calorimeter cell containing 25 μM MAP2c N-MAP2c or MAP2c 159 to 467 to achieve a complete binding isotherm. The heat of dilution was measured by injecting RIIα-PKA or RIIDD_2_ into the buffer solution. Blank measurement was measured by titrating buffer into the cell containing MAP2c or N-MAP2c. For competitive ITC, the cell contained 25 μM MAP2c phosphorylated by Fyn with 100 μM Grb2. The heat of dilution and the blank measurement were subtracted from the heat of reaction to obtain the effective heat of binding. Titration curves were fitted using the MicroCal PEAQ-ITC software, assuming one set of binding sites.

### NMR spectroscopy

NMR experiments were measured on 850 MHz and 950 MHz NMR Bruker Avance III HD spectrometers equipped with 5 mm quadruple-resonance (^1^H-^13^C-^15^N-^31^P) and triple-resonance (^1^H-^13^C-^15^N), respectively, inverse cryoprobes with cooled ^1^H and ^13^C preamplifiers and with *z*-axis gradients, except for HCCH-TOCSY spectra recorded at 600 MHz Bruker Avance III HD spectrometers equipped with a 5 mm quadruple-resonance (^1^H-^13^C-^15^N-^31^P) inverse cryoprobe with cooled ^1^H and ^13^C preamplifiers and with *z*-axis gradients. The temperature was calibrated using a standard sample of neat methanol and set to 27 °C. All NMR measurements were performed in 50 mM MOPS, 100 mM NaCl, 0.5 mM TCEP, pH 6.9, containing 10% deuterium oxide. The ^1^H carrier frequencies were set to the water resonance (4.7 ppm) in all experiments.

The 3D HNCO (53) spectra were acquired on samples containing 0.1 mM [^13^C,^15^N]-MAP2c with spectral widths set to 2000 Hz and 2000 Hz, maximal evolution times of 120 ms and 80 ms in the ^15^N and ^13^C indirectly detected dimensions, respectively. The ^15^N and ^13^C carrier frequencies were set to 118.0 ppm and 174.7 ppm, respectively. The overall number of 2048 complex points was acquired in the acquisition dimension, and 2000 hypercomplex points were randomly distributed over the indirectly-detected dimensions. Number of scans per FID was 8. 2D ^1^H,^15^N HSQC spectra were typically acquired with four scans, with the spectral width of 21 ppm or 30 ppm and 64 to 256 complex points in the indirect dimension and with the ^15^N carrier frequency of 118.0 ppm.

All triple resonance and ^15^N-edited NOESY experiments for the assignment of the N-MAP2c:RIIDD_2_ complex were recorded with the ^15^N spectral width of 21 ppm, with the ^15^N carrier frequency of 117.6 ppm and with 20 and 64/112 complex points in the ^15^N and ^13^C/^1^H indirectly detected dimensions, respectively. The number of scans was 16 unless specified otherwise. The mixing time in all NOESY experiments was 120 ms.

The triple resonance and ^15^N-edited NOESY spectra of a sample containing 0.5 mM [^13^C,^15^N]-N-MAP2c and 2 mM unlabeled RIIDD_2_ and of a sample containing 1.2 mM unlabeled N-MAP2c and 0.6 mM [^13^C,^15^N]-RIIDD_2_ (expressed for monomeric units of RIIDD_2_) were recorded at 950 MHz with the following ^13^C or ^1^H spectral widths and carrier frequencies, respectively. HNCA and HN(CO)CA (8 scans): 30 ppm and 55.9 ppm, HNCACB (24 scans) and CBCA(CO)NH: 30 ppm and 55.9 ppm, HNCO: 14 ppm and 176.2 ppm, ^15^N-edited TOCSY: 16.4 ppm and 4.7 ppm, 64 complex points, mixing time of 60 ms, ^15^N-edited NOESY: 13.8 ppm and 4.7 ppm. HCCH-TOCSY spectra of the same samples were acquired at 600 MHz with the ^13^C carrier frequency of 45.7/125.7 ppm, with 32/16 complex points and spectral width set to 80/40 ppm in the ^13^C indirectly detected dimension, with 64 complex points and spectral width set to 10 ppm in the ^1^H indirectly detected dimension and with the mixing time of 2.7 ms.

The triple resonance and ^15^N-edited NOESY spectra of a sample containing 0.45 mM N-MAP2c labeled with [^13^C,^15^N]-Ile,Val and [^15^N]-Leu and 1.8 mM unlabeled RIIDD_2_ and of samples containing 1.2 mM unlabeled N-MAP2c and 0.6 mM [^13^C,^15^N]-RIIDD (expressed for monomeric units of RIIDD) were recorded at 850 MHz with the following ^13^C or ^1^H spectral widths and carrier frequencies, respectively. HNCA and HN(CO)CA: 21 ppm and 59 ppm, HNCO: 14 ppm and 176.2 ppm, ^15^N-edited NOESY: 14 ppm and 4.7 ppm. A ^13^C-edited NOESY spectrum of the same sample was acquired with the ^13^C carrier frequency of 41.7 ppm, with spectral width set to 65 ppm and 10.5 ppm and with 20 and 112 complex points in the ^13^C and ^1^H indirectly detected dimensions, respectively. An HCCH-TOCSY spectrum of the same samples was acquired at 600 MHz with the ^13^C carrier frequency of 45.7 ppm, with 32 complex points and spectral width set to 80 ppm in the ^13^C indirectly detected dimension, with 64 complex points and spectral width set to 10 ppm in the ^1^H indirectly detected dimension, and with the mixing time of 2.7 ms.

In addition to the ^15^N-edited NOESY spectrum described above, the following 3D NOESY spectra for structure determination of the N-MAP2c:RIIDD_2_ complex we recorded at 950 MHz on the sample containing 0.5 mM [^13^C,^15^N]-N-MAP2c and 2 mM unlabeled RIIDD_2_ and on the sample containing 1.2 mM unlabeled N-MAP2c and 0.6 mM [^13^C,^15^N]-RIIDD_2_ (expressed for monomeric units of RIIDD): ^13^C-edited NOESY spectra with the ^13^C carrier frequencies of 45.7/126.2 ppm, with spectral widths set to 80/30 ppm and 13.8/13.8 ppm and with 32/16 and 128/100 complex points in the ^13^C and ^1^H indirectly detected dimensions, respectively; and ^15^N-edited, ^13^C,^15^N-filtered NOESY spectra (32 scans) with the ^15^N carrier frequencies of 117.6 ppm, with spectral widths set to 21 ppm and 13.8 ppm and with 32 and 72 complex points in the ^15^N and ^1^H indirectly detected dimensions, respectively. The following spectra were recorded on the sample containing 0.5 mM [^13^C,^15^N]-N-MAP2c and 2 mM unlabeled RIIDD_2_ only: ^13^C-edited, ^13^C-filtered NOESY spectrum (32 scans) with the ^13^C carrier frequencies of 45.7 ppm, with spectral widths set to 80 ppm and 13.8 ppm and with 32 and 72 complex points in the ^13^C and ^1^H indirectly detected dimensions, respectively; and ^13^C-edited, ^13^C,^15^N-filtered NOESY spectrum (32 scans) with the ^13^C carrier frequencies of 45.7 ppm, with spectral widths set to 80 ppm and 10 ppm and with 32 and 48 complex points in the ^13^C and ^1^H indirectly detected dimensions, respectively.

2D ^1^H,^15^N HSQC spectra that were acquired to follow the phosphorylation of ^15^N-tyrosine-labeled MAP2c by Fyn or Abl were recorded with 16 scans, with the spectral width of 14 ppm and 2048 complex points in the acquisition dimension, the spectral width of 26 ppm and 128 complex points in the indirectly detected dimension, and with the ^15^N carrier frequency of 122 ppm.

2D ^1^H,^13^C HMQC spectra used to track the intensity of the peak corresponding to pTyr ^13^Cε-Hε during Fyn phosphorylated samples preparation were recorded with 16 scans, with the spectral width of 14 or 23 ppm and 2048 complex points in the acquisition dimension, the spectral width of 30 ppm and 128 complex points in the indirectly detected dimension, and with the ^13^C carrier frequency of 125 ppm.

2D ^1^H,^15^N HSQC spectra of Grb2-SH2 were recorded with 400 scans, with the spectral width of 13 ppm, and 2048 complex points in the acquisition dimension, the spectral width of 30 ppm and 128 complex points in the indirectly detected dimension, and with the ^15^N carrier frequency of 118 ppm.

^1^H,^15^N HSQC-TOCSY spectra of a sample containing 0.35 mM [^15^N]-Grb2-SH2 were recorded with 16 scans, the spectral width of 12 ppm and 2048 complex points in the acquisition dimension, the spectral width of 12 ppm and 200 complex points in ^1^H indirectly detected dimension, the spectral width of 24 ppm and 40 complex points in the ^15^N indirectly detected dimension, and ^15^N carrier frequency of 120.5 ppm.

The ^1^H,^15^N HSQC spectrum of [^15^N]-Grb2-SH2 was assigned using chemical shifts published by Wang *et al.* (92), Penk *et al.* (75), Thornton *et al.* (93), Sanches *et al.* (94) (BMRB-ID: 27781), and Ogura *et al.* (76) (BMRB-ID:11055) and verified by ^1^H,^15^N HSQC-TOCSY.

2D ^1^H,^15^N HSQC of 0.2 mM [^15^N]-MAP2c 300 to 467 with 0.1, 0.2, 0.4, 0.8, and 1.6 mM RIIDD_2_ were recorded with 16 scans, with the spectral width of 14 ppm and 2048 complex points in the acquisition dimension, the spectral width of 26 ppm and 128 complex points in the indirectly detected dimension, and with the ^15^N carrier frequency of 122 ppm.

### Residual dipolar coupling measurement

Magnetically oriented phage *Pf1* (Asla Biotech) was dialyzed into NMR buffer. Oriented sample contained 733 μl 0.1 mM [^15^N] N-MAP2c, 0.4 mM unlabeled RIIDD_2_, 40 mg·ml^−1^ *Pf1*, 50 mM MOPS, 100 mM NaCl, 8% D_2_O, pH 6.9 in a 5 mm NMR tube. The isotropic sample contained 400 μl 0.23 mM [^15^N] N-MAP2c, 0.92 mM unlabeled RIIDD_2_, 50 mM MOPS, 100 mM NaCl, 7.7% D_2_O, pH 6.9, in a 5 mm Shigemi NMR tube. The ^1^H-^15^N HSQC spectra (54, 55) were acquired with 2048 and 256 real points in the direct and indirect dimensions, respectively. 2D in-phase, anti-phase spectra (66) were acquired with 2048 and 512 real points in the direct and indirect dimensions, respectively. Peak picking was done in each of the four subspectra (IP and AP oriented, IP and AP isotropic) using previously assigned ^1^H-^15^N HSQC spectra. RDC values were calculated from frequencies in the ^15^N dimension as ^1^*D*_NH_=(δ_IP,oriented_ − δ_AP,oriented_) − (δ_IP,isotropic_ − δ_AP,isotropic_). The values were analyzed and fitted against calculated values of 25 ranked structures from AlphaFold 2.2.0 (67) using an in-house written program dconv 0.8.9.

### *In vitro* phosphorylation

^15^N-tyrosine-labeled MAP2c was dialyzed overnight into NMR buffer (50 mM MOPS, 100 mM NaCl, 0.5 mM TCEP, pH 6.9). To follow phosphorylation by Fyn, the protein was diluted in NMR buffer containing 10 mM ATP, 10 mM MgCl_2_, 0.1 mM EDTA, and 10% D_2_O to a final concentration of 100 μM and series of 2D ^1^H,^15^N HSQC spectra were measured in the intervals of 45 min at 27 °C after addition of 4.8 μg of Fyn kinase (Fyn Protein, active, Sigma-Aldrich).

To follow phosphorylation by Abl, ^15^N-tyrosine-labeled MAP2c in NMR buffer containing 10 mM ATP, 10 mM MgCl_2_, 0.1 mM EDTA, and 10% D2O was mixed with 296 μg of Abl. Final concentration of MAP2c in the NMR sample was 190 μM. Series of 2D ^1^H,^15^N HSQC spectra were measured in intervals of 45 min at 27 °C.

[^13^C^15^N]-MAP2c for HNCO NMR experiments was dialyzed into NMR buffer, concentrated, and diluted into NMR buffer containing 20 mM ATP, 20 mM MgCl_2_, 0.1 mM EDTA, and 10% D_2_O to a final concentration of 790 μM in a final volume of 500 μl. Upon addition of 10 μg of Fyn kinase (Fyn Protein, active, Sigma-Aldrich), the phosphorylation was monitored as the intensity of peak corresponding to pTyr ^13^Cε-^1^Hε in a series of 2D ^1^H,^13^C HMQC spectra acquired in intervals of 53 min at 27 °C. After plateau was reached, the sample was diluted 20 × with 50 mM sodium acetate, 1 mM EGTA, 1 mM MgCl_2_, 4 mM β-mercaptoethanol, pH 5.5, loaded to a HiTrap SP HP column (Cytiva), eluted with a gradient of 1 M NaCl (0–50%), concentrated, and dialyzed to NMR buffer.

Based on the ratio of volumes of well-resolved peaks in the 3D HNCO spectra corresponding to residues of Fyn-phosphorylated and unphosphorylated [^13^C,^15^N]-MAP2c with distinct chemical shifts (Gly65–Asp69 except for Tyr67), the level of phosphorylation was estimated to be 75%.

Unlabeled MAP2c phosphorylated by Fyn for CSP analysis was prepared in the same way as described above for [^13^C^15^N]-MAP2c except of the monitoring of phosphorylation *via* NMR. The sample was incubated with Fyn at 27 °C for 61 h 30 min. The time span was based on previous experience with phosphorylation monitored *via* NMR.

### CSP analysis

CCSP for [^15^N]-SH2-Grb2 (^1^H-^15^N HSQC spectrum) was calculated as (95):

$$CCSP=\sqrt{\frac{1}{2}\left[\Delta \delta_{H}^{2}+{\left(\alpha_{N}\Delta \delta_{N}\right)}^{2}\right]}$$

where $\Delta \delta_{H}$ is chemical shift change in ^1^H (ppm), $\Delta \delta_{N}$ is chemical shift change in ^15^N (ppm). For scaling factor $\alpha_{N}$, the value 0.16 was used.

CCSP for [^13^C,^15^N]-MAP2c (HNCO spectrum) was calculated as:

$$CCSP=\sqrt{\frac{1}{3}\left[\Delta \delta_{H}^{2}+{\left(\alpha_{N}\Delta \delta_{N}\right)}^{2}+{\left(\alpha_{C}\Delta \delta_{C}\right)}^{2}\right]}$$

where $\Delta \delta_{H}$ is chemical shift change in ^1^H (ppm), $\Delta \delta_{N}$ is chemical shift change in ^15^N (ppm), and $\Delta \delta_{C}$ is chemical shift change in ^13^C (ppm). For the scaling factors $\alpha_{N}$ and $\alpha_{C}$, the values 0.16 and 0.045 were used, respectively.

Dissociation constants *K*_D_ were obtained by fitting CCSP to the equation

$$y=\frac{a}{2p}\left[c+p+K_{D}-\sqrt{{\left(c+p+K_{D}\right)}^{2}-4cp}\right]$$

where *y* is CCSP at the total concentration *c* of the unlabeled protein, *p* is the total concentration of the observed isotope-labeled protein, and *a* is the CCSP value corresponding to completely bound isotope-labeled protein.

### NMR structure calculation

CYANA 3.98.15 (68) was used for automated NOE assignment and structure calculation. Input files and control scripts were based on publicly available templates from CYANA wiki (cyana.org). The scripts can be found in Supporting information (Table S2). Torsion angles were predicted using TALOS-N 4.12 (69).

CNS 1.21 (70) with SculptorCNS 1.21-3.1 (https://www.ibs.fr/IMG/pdf/sculptorCns_documentation.pdf) was used for structure refinement. This software uses a full Lennard-Jones potential and TIP3P explicit water model. Scripts from the Recoord database (71) were used to control CNS.

### Molecular dynamics simulation

The molecular dynamics simulations were performed using the GROMACS package, under the 2022.3 version (96, 97). The revised CHARMM36m (98) protein force field was used to parametrize the protein, while parameters for the solvent environment were taken from the CGenFF version 4.6 (99, 100), including the TIP3P (101) water model parameters. The PDB entry 1QG1 (76) was used as a basis for the initial system coordinates. In order to employ periodic boundary conditions, each system was placed into a truncated octahedral (triclinic) box of the volume of 320.37 nm^3^, corresponding to a box vector length of 7.466 nm. A larger box was used for the modified peptide system to avoid periodic image contacts due to its increased flexibility. A vector of 15 nm was used, corresponding to a volume of 2598.08 nm^3^. Sodium chloride was added to each system, corresponding to the concentration of 155 mmol dm^−3^.

Nonbonding interactions were addressed by the following setup: long-range electrostatic interactions were calculated by particle mesh Ewald summation (102). The cutoff for accompanying short-range electrostatic interactions was set to 1.2 nm. Van der Waals interactions were calculated using Lennard-Jones potential, with a cutoff of 1.2 nm. These were further supplemented by a long-range dispersion correction for energy and pressure, as well as the switching of forces to zero at the cutoff distance. Additionally, bonds involving hydrogen atoms were constrained using the LINCS algorithm (103) after the energy minimization step. The temperature and pressure, which were introduced during the respective equilibration steps, were maintained using the velocity rescaling thermostat (104) and Parrinello-Rahman barostat (105) algorithms. Protein atoms were treated as a separate group for the purpose of temperature coupling, so there were two groups in total with a coupling time constant of 0.1 ps each. The time constant for pressure coupling was set to 40 ps and the system compressibility was set to 4.5 × 10^−10^ Pa. See the appropriate GROMACS documentation (106) for details, as well as the default values which were used for unspecified parameters.

Both systems were subjected to the same equilibration and data collection procedure. First, a solvent energy minimization was carried out using the steepest descent algorithm. A 100 ps NVT equilibration followed at 310 K with restrained protein backbone coordinates (1000 kJ mol^−1^ nm^−2^) and a time step of 1 fs. Additional 200 ps of NPT equilibration were performed at 310 K and 1 bar, with the same restriction and time step. Finally, the restriction was removed, time step was set to 2 fs and 50 ns worth of trajectory were collected under the same conditions, with a sampling frequency of 10 ps.

## Data availability

The structural and NMR data are deposited in the PDB and BMRB databases, ID 8S8O and 34908, respectively. Other data are presented in the article.

## Supporting information

This article contains supporting information.

## Conflict of interest

The authors declare that they have no conflicts of interest with the contents of this article.
