# Supplementary material for: Structural basis of binding the unique N-terminal domain of microtubule-associated protein 2c to proteins regulating kinases of signaling pathways
Source: J Biol Chem. 2024 Jul 11;300(8):107551. doi: 10.1016/j.jbc.2024.107551 (PMC11367651; doi:10.1016/j.jbc.2024.107551)
Supplement: Supporting Information [file mmc1.pdf]

SUPPORTING INFORMATION

Structural basis of binding the unique N-terminal domain of microtubule-associated protein 2c to proteins regulating kinases of signaling pathways

Viktor Bartošík[1,*], Jitka Plucarová[1,2,*], Alice Laníková[1,2], Zuzana Janáčková[1], Petr Padrta[1,2], Séverine Jansen[1,2], Vojtěch Vařečka[3], Tobias Gruber[4,5], Stephan M. Feller[4], Lukáš Žídek[1,2]

[1]National Centre for Biomolecular Research, Faculty of Science, Masaryk University, Kamenice 5, CZ-625 00 Brno, Czech Republic

[2]Central European Institute of Technology, Masaryk University, Kamenice 5, CZ-625 00 Brno, Czech Republic

[3]Institute of Chemistry, Faculty of Science, Masaryk University, Kamenice 5, CZ-625 00 Brno, Czech Republic

[4]Institute of Molecular Medicine, Tumor Biology, Martin-Luther-University of Halle-Wittenberg, Germany

[5]Institute of Physics, Biophysics, Martin-Luther-University of Halle-Wittenberg, Germany

*V.B. and J.P. contributed equally

correspondence: Lukáš Žídek, lzidek@chemi.muni.cz

Contents

Table S1. Alignment of selected vertebrate Grb2 SH2 binding sequences of EGFR and CD28.

Table S2. The CYANA running script CALC.cya and additional settings in init.cya

Figure S1. Results of ITC analysis of MAP2c titrated by RIIα-PKA and $RIIDD_2$.

Figure S2. Interactions of MAP2c fragments consisting of residues 159–467 and 300–467 with RIIα-PKA.

Figure S3. Comparison of structural models of the N-MAP2c:$RIIDD_2$ complex predicted by AlphaFold multimer and AlphaFold 3 (B), and of a model of the binding region of free MAP2c predicted by AlphaFold 2 with the representative structure calculated in our study.

Figure S4. Overlay of ^{1}H-^{15}N HSQC spectra of selectively [^{15}N-Tyr] labeled MAP2c before and after phosphorylation by Fyn and Abl, and changes in the tyrosine peak heights in the course of Fyn and Abl phosphorylation.

Figure S5. Combined chemical shift perturbations (CCSP) of unphosphorylated MAP2c upon addition Grb2-SH2 and of Grb2-SH2 upon addition of unphosphorylated MAP2c.

Figure S6. Snapshots of molecular dynamics simulations of phosphopeptides containing the pTyr-Val-Asn-Val sequence and the pTyr-Ser-Asp-Thr sequence in complex with Grb2-SH2 and distances between phosphopeptide pTyr and Arg86 of Grb2-SH2.

Figure S7. Results of ITC analysis of Fyn-phosphorylated MAP2c titrated by RIIα-PKA in absence and presence of 100 μM Grb2.

Table S1. Alignment of selected vertebrate Grb2 SH2 binding sequences of EGFR and CD28. Tyrosines corresponding to human MAP2 Tyr67 are in bold, asparagines aligned with human MAP2 Asn69 are shown in green.

EGFR:

**Class**	**Order**	**Binomial name**	**Sequence**
Mammalia	Primates	*Homo sapiens*	FLPVPE**Y**INQSVPKR
Mammalia	Primates	*Macaca fascicularis*	FLPVPE**Y**INQSVPKR
Mammalia	Rodentia	*Rattus norvegicus*	FLPVPE**Y**INQSVPKR
Mammalia	Lagomorpha	*Lepus europaeus*	FLPVPE**Y**INQYIPKR
Mammalia	Chiroptera	*Pteropus giganteus*	FLPVPE**Y**INQSIPKR
Mammalia	Chiroptera	*Myotis myotis*	FLPAPE**Y**INQSVPKR
Mammalia	Carnivora	*Lynx canadensis*	FLPAPE**Y**INQAVPKR
Mammalia	Artiodactyla	*Sus scrofa*	FLPAPE**Y**VNQSVPKR
Mammalia	Artiodactyla	*Cervus canadensis*	FLPAPE**Y**VNQSVPKR
Mammalia	Diprotodontia	*Phascolarctos cinereus*	FQPVPE**Y**INQSAPKN
Mammalia	Monotremata	*Ornithorhynchus anatinus*	FLPVPE**Y**INQSAPQR
Aves	Passeriformes	*Serinus canaria*	FLPAPE**Y**VNQLVPKK
Aves	Galliformes	*Phasianus colchicus*	FLPAPE**Y**VNQLMPKK
Aves	Falconiformes	*Falco rusticolus*	FLPAPE**Y**VNQLAPKK
Aves	Columbiformes	*Columba livia*	FLPAPE**Y**VNQLVPKK
Aves	Casuariiformes	*Dromaius novaehollandiae*	FLPAPE**Y**VNQLMPKK
Reptilia	Squamata	*Anolis carolinensis*	FLPAPE**Y**MNQLAAKP
Reptilia	Testudines	*Caretta caretta*	FLPAPE**Y**VNQSIPKK
Amphibia	Gymnophiona	*Geotrypetes seraphini*	FLPAPE**Y**VNETHPEK
Amphibia	Anura	*Bufo bufo*	FHPIPE**Y**VNETNSKP
Actinopterygii	Polypteriformes	*Erpetoichthys calabaricus*	FQPAAE**Y**VNQNGQTM
Actinopterygii	Lepisosteiformes	*Lepisosteus oculatus*	FQPVPE**Y**VNQNGNPV (note: predicted)
Actinopterygii	Cypriniformes	*Danio rerio*	APGYNE**Y**MNQNESSM

Table S1. continued

CD28:

**Class**	**Order**	**Binomial name**	**Sequence**
Mammalia	Primates	*Homo sapiens*	RLLHSD**Y**MNMTPRRP
Mammalia	Primates	*Macaca fascicularis*	RLLHSD**Y**MNMTPRRP
Mammalia	Rodentia	*Rattus norvegicus*	RLLQSD**Y**MNMTPRRL
Mammalia	Lagomorpha	*Lepus europaeus*	RLLQSD**Y**MNMTPRRP
Mammalia	Chiroptera	*Pteropus giganteus*	RILQSD**Y**MNMTPRRP
Mammalia	Chiroptera	*Myotis myotis*	RVLQSD**Y**LNMTPRRL
Mammalia	Carnivora	*Lynx canadensis*	RILQSD**Y**MNMTPRRP
Mammalia	Artiodactyla	*Sus scrofa*	RMLQSD**Y**MNMTPRRL
Mammalia	Artiodactyla	*Cervus canadensis*	RMLQSD**Y**MNMTPRRP
Mammalia	Diprotodontia	*Phascolarctos cinereus*	RILHSD**Y**MNMTPRRP
Mammalia	Monotremata	*Ornithorhynchus anatinus*	RILQSD**Y**MNMTPRRP
Aves	Passeriformes	*Serinus canaria*	MYHQSD**Y**MNMTPRHP
Aves	Galliformes	*Phasianus colchicus*	RYRQSD**Y**MNMTPRHP
Aves	Falconiformes	*Falco peregrinus*	MYHQSD**Y**MNMTPRHP
Aves	Columbiformes	*Columba livia*	MYHQSD**Y**MNMTPRHP
Aves	Casuariiformes	*Dromaius novaehollandiae*	MYHQSD**Y**MNMTPRHP
Reptilia	Squamata	*Anolis carolinensis*	RIVRND**Y**FNMTPWQS
Reptilia	Testudines	*Caretta caretta*	RILTSD**Y**MNMTPRHP
Amphibia	Gymnophiona	*Rhinatrema bivittatum*	RILQSD**Y**MNMTPRRP
Amphibia	Anura	*Bufo bufo*	QIQQSE**Y**INVVPRRP<br>(note: CD28-like)
Actinopterygii	Polypteriformes	*Erpetoichthys calabaricus*	KQMQND**Y**MNMPSKQK<br>(note: CD28-like)
Actinopterygii	Lepisosteiformes	*Lepisosteus oculatus*	KHNQND**Y**MNMKPRGL<br>(note:predicted, CD28-like)
Actinopterygii	Cypriniformes	*Danio aesculapii*	missing<br>(note: CD28-like)

Table S2. The CYANA running script CALC.cya and additional settings in init.cya

```
#the running script "CALC.cya"
peaks       :=
MAP2c_NNEW.peaks,MAP2c_aliNEW.peaks,RIIDD_N.peaks,RIIDD_ali.peaks,MAP2c_filtered_ali.p
eaks,MAP2c_filtered_N.peaks,RIIDD_filtered_ali.peaks,RIIDD_filtered_N.peaks # NOESY peak lists
in XEASY format
prot        :=  MAP2c_RIIDD_NEW.prot                  # names of chemical shift list(s)
restraints  :=  talosn_NEW.aco,MAP2c.rdc,artificial.upl,artificial.lol            # additional (non-NOE)
restraints
tolerance   := 0.033, 0.021, 0.45              # shift tolerances: H, H', C/N', C/N
upl_values := 2.4,6
#calibration_dref := 4
structures  := 100,20                      # number of initial, final structures
steps       := 10000                       # number of torsion angle dynamics steps
randomseed  := 434726                       # random number generator seed
weight_rdc   = 0.4               # weight for RDC restraints
cut_rdc      = 3                # cutoff for RDC violation output
noeassign peaks=$peaks prot=$prot autoaco

#additional settings in "init.cya"
read cyana.lib
read MAP2c_RIIDD_short.seq
rmsdrange:=8-49,108-149,286-309
nproc=4
cut_upl=0.5
cut_lol=0.5
hbond alpha=13-29 weight=1
hbond alpha=33-49 weight=1
hbond alpha=113-129 weight=1
hbond alpha=133-149 weight=1
hbond alpha=286-310 weight=1
```

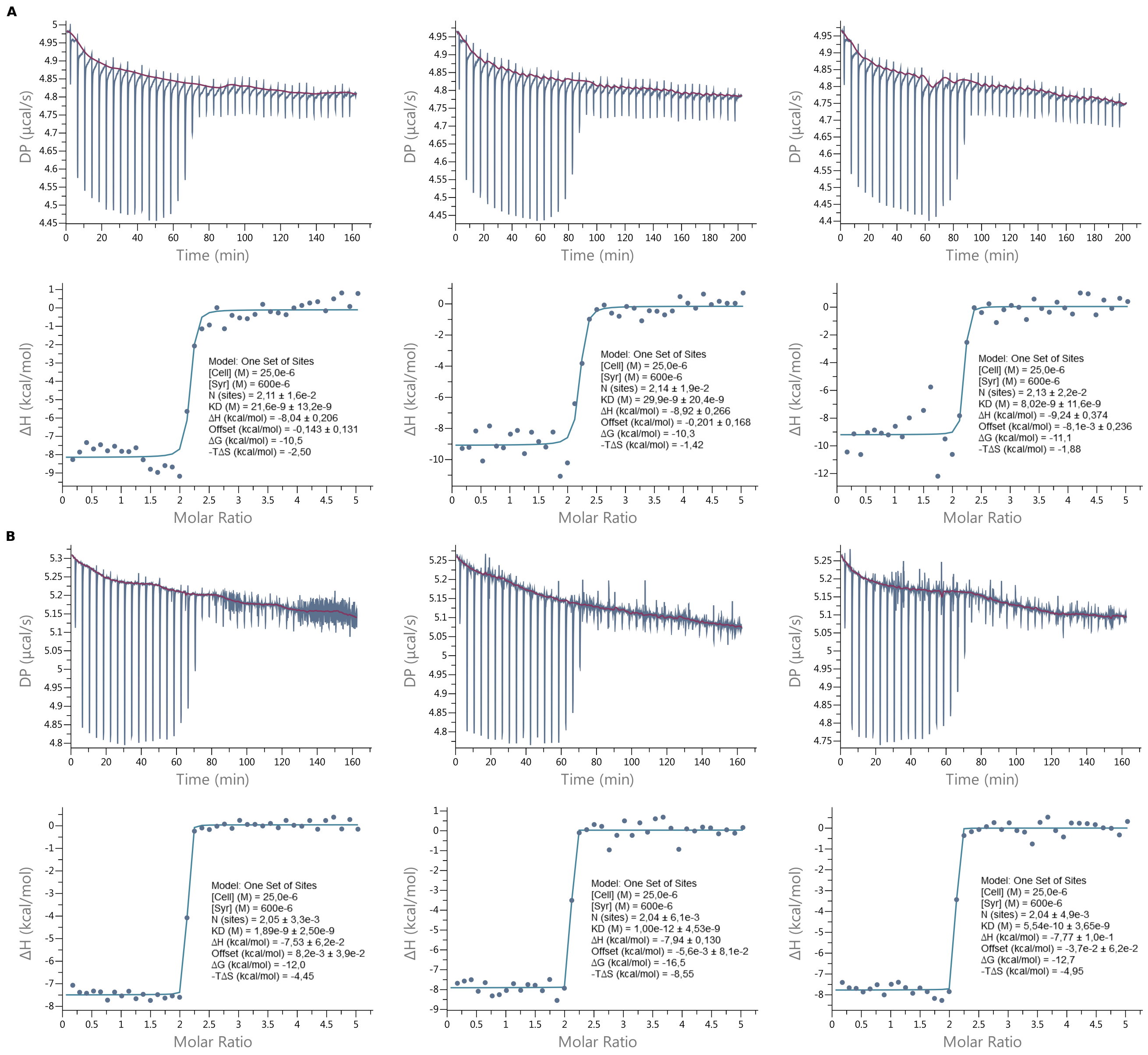

Figure S1. Results of ITC analysis of MAP2c titrated by RIIα-PKA (A) and $RIIDD_2$ (B). The concentration of MAP2c and N-MAP2c in the cell was 25 µM, the concentration of RIIα-PKA and $RIIDD_2$ in the syringe was 600 µM. Results of the fits in the insets document that stoichiometry and enthalpy of the binding was determined reproducibly, but the binding isotherms are too steep for reliable estimation of K_D.

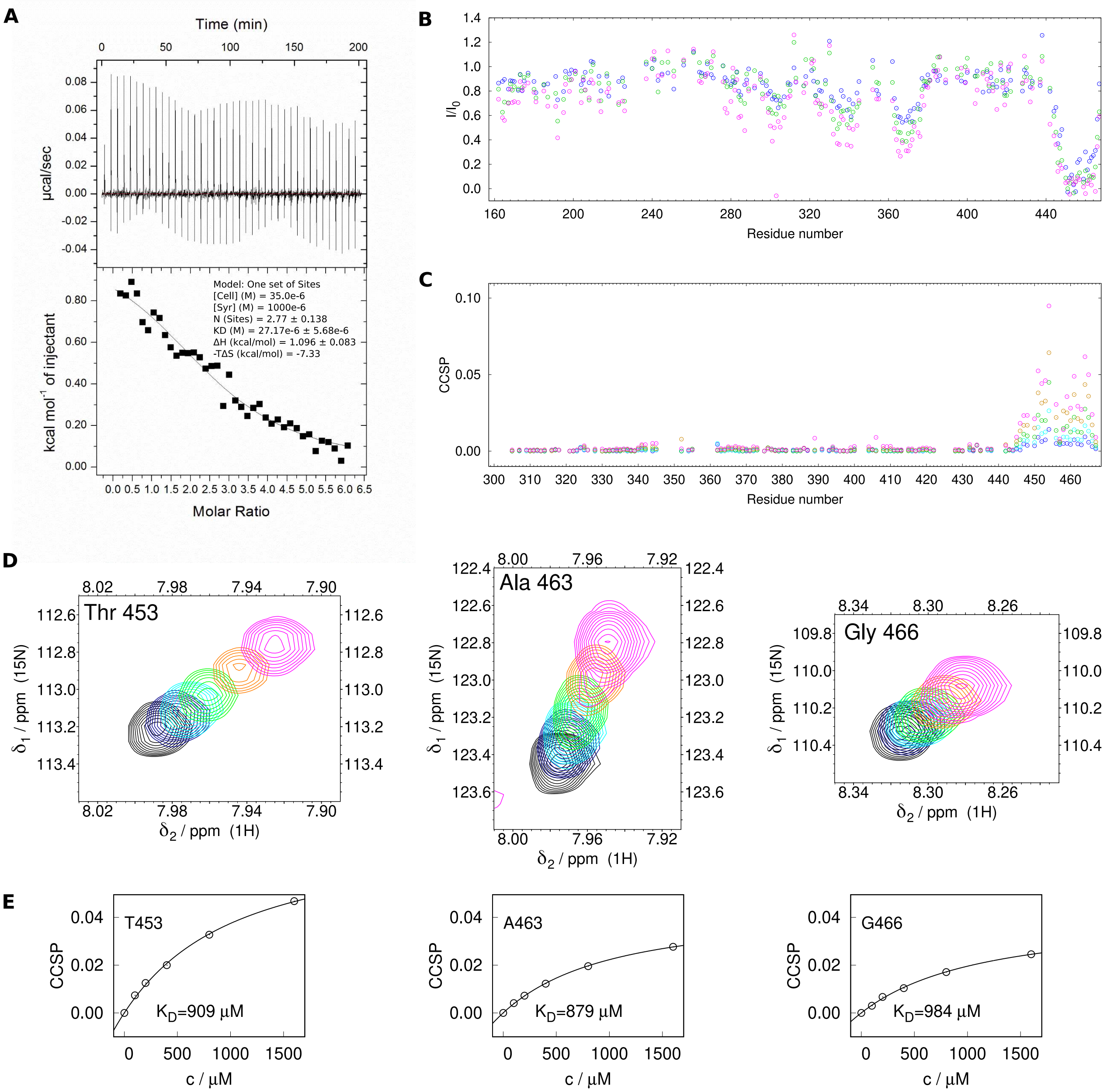

Figure S2. A, Results of ITC measurements of MAP2c fragment consisting of residues 159–467, revealing a relatively weak binding (K_D of 27 μM with ±6 μM error of fitting). B, NMR peak broadening of 100 μM [^{13}C,^{15}N] MAP2c fragment consisting of residues 159–467 in presence of 100 μM (blue), 200 μM (green), and 400 μM (magenta) RIIα-PKA. Decreasing peak height I/I_0 identified interactions of RIIα-PKA with proline-rich region P2 (residues Ser280–Leu300) and in the microtubule-binding repeat 3 (Val333–Arg363), but especially with the C-terminus of the fragment. C, combined chemical shift perturbations of 200 μM [^{15}N] MAP2c fragment consisting of residues 300–467 in presence of 100 μM (blue), 200 μM (cyan), 400 μM (green), 800 μM (orange), and 1600 μM (magenta) RIIα-PKA. The values revealed binding of RIIα-PKA exclusively to the C-terminal helical region of MAP2c. Spectra of selected peaks (color-coded as in Panel C, black spectra were recorded in absence of RIIα-PKA) and fitting of their CCSP are presented in Panels D and E, respectively. The estimated K_D of ~0.9 mM indicated very weak binding.

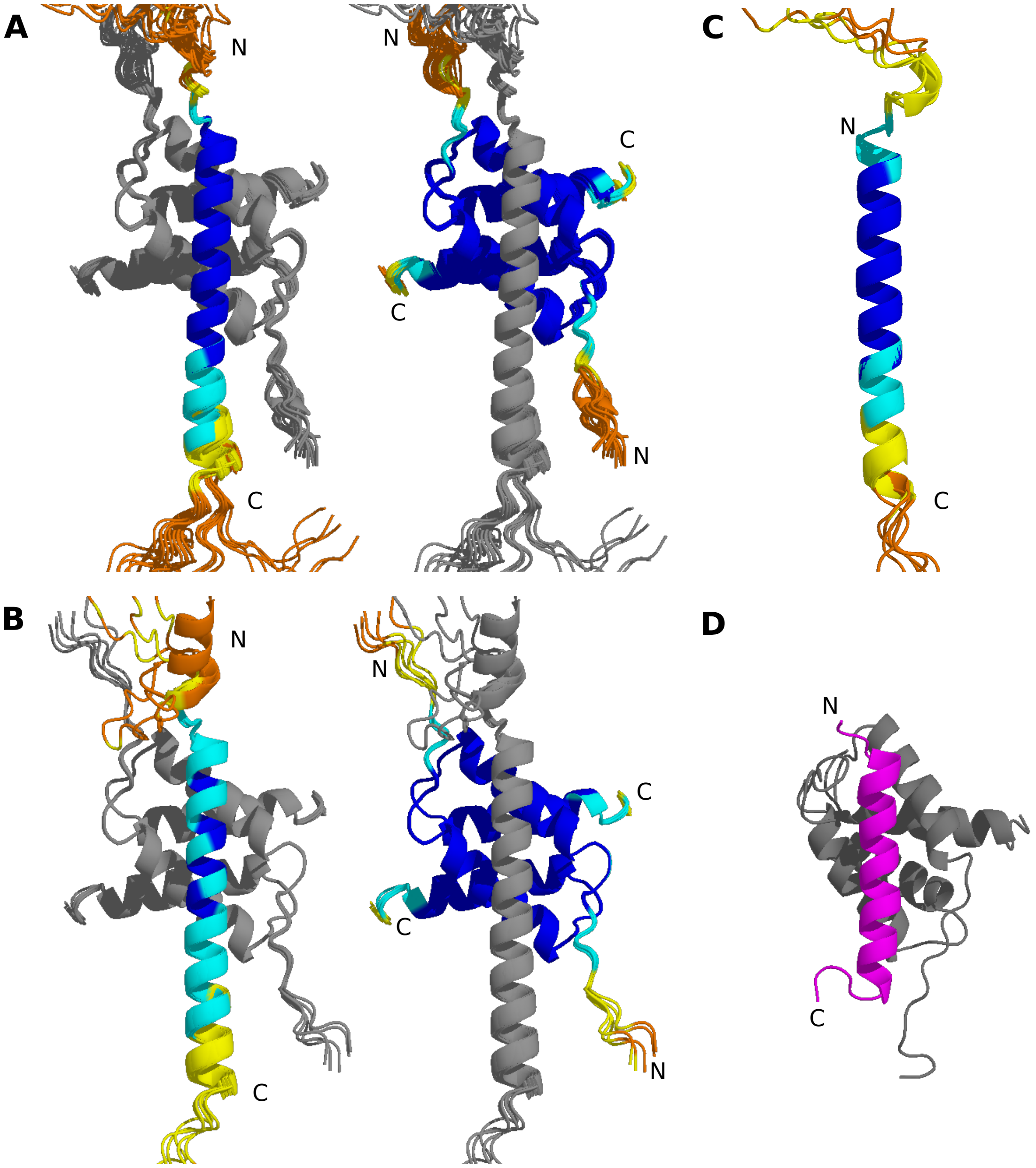

Figure S3 Comparison of structural models of the N-MAP2c:$RIIDD_2$ complex predicted by AlphaFold multimer (A) and AlphaFold 3 (B), of a model of the binding region of free MAP2c predicted by AlphaFold 2 (C), and of the representative structure calculated in our study (D). The structures predicted by AlphaFold are colored (separately for N-MAP2c and $RIIDD_2$) according to the AlphaFold confidence score: blue, pLDDT>90, cyan, 90>pLDDT>70, yellow, 70>pLDDT>50, and red, pLDDT<50. In the experimental structure, N-MAP2c and $RIIDD_2$ are shown in magenta and gray, respectively.

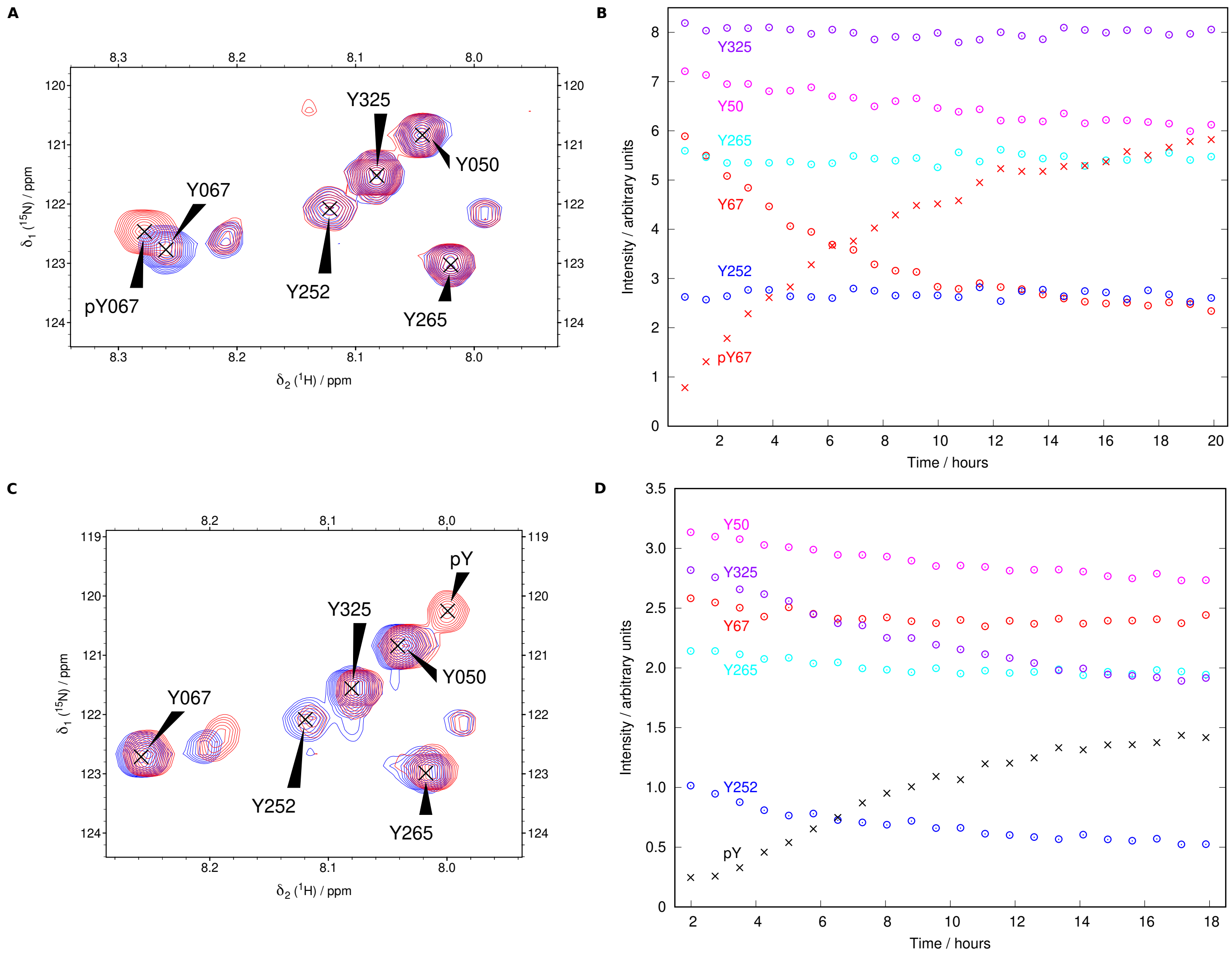

Figure S4 A,C, Overlay of ^{1}H-^{15}N HSQC spectra of selectively [^{15}N-Tyr] labeled MAP2c before (blue) and after (red) phosphorylation by Fyn (A) and (C) Abl kinases. B,D, Changes in the tyrosine peak heights in the course of phosphorylation by Fyn (B) and (D). Abl kinases The spectra were measured for 20 hrs upon addition of the kinase. MAP2c specifically ^{15}N-labeled on tyrosines was used because ^{1}H,^{15}N HSQC spectrum of uniformly labeled MAP2c is not sufficiently resolved in the region of the tyrosine peaks. This approach allowed us to observe changes at all tyrosines directly, simultaneously, and in a real time. Peak heights of unphosphorylated Tyr50 (magenta), Tyr67 (red), Tyr252 (blue), Tyr265 (cyan), and Tyr325 (purple) are plotted as circles. Peak heights of phosphorylated pTyr67 (red) and of an unassigned pTyr phosphorylated by Abl (black) are plotted as crosses. The results confirm that Fyn phosphorylates selectively Tyr67 of MAP2c.

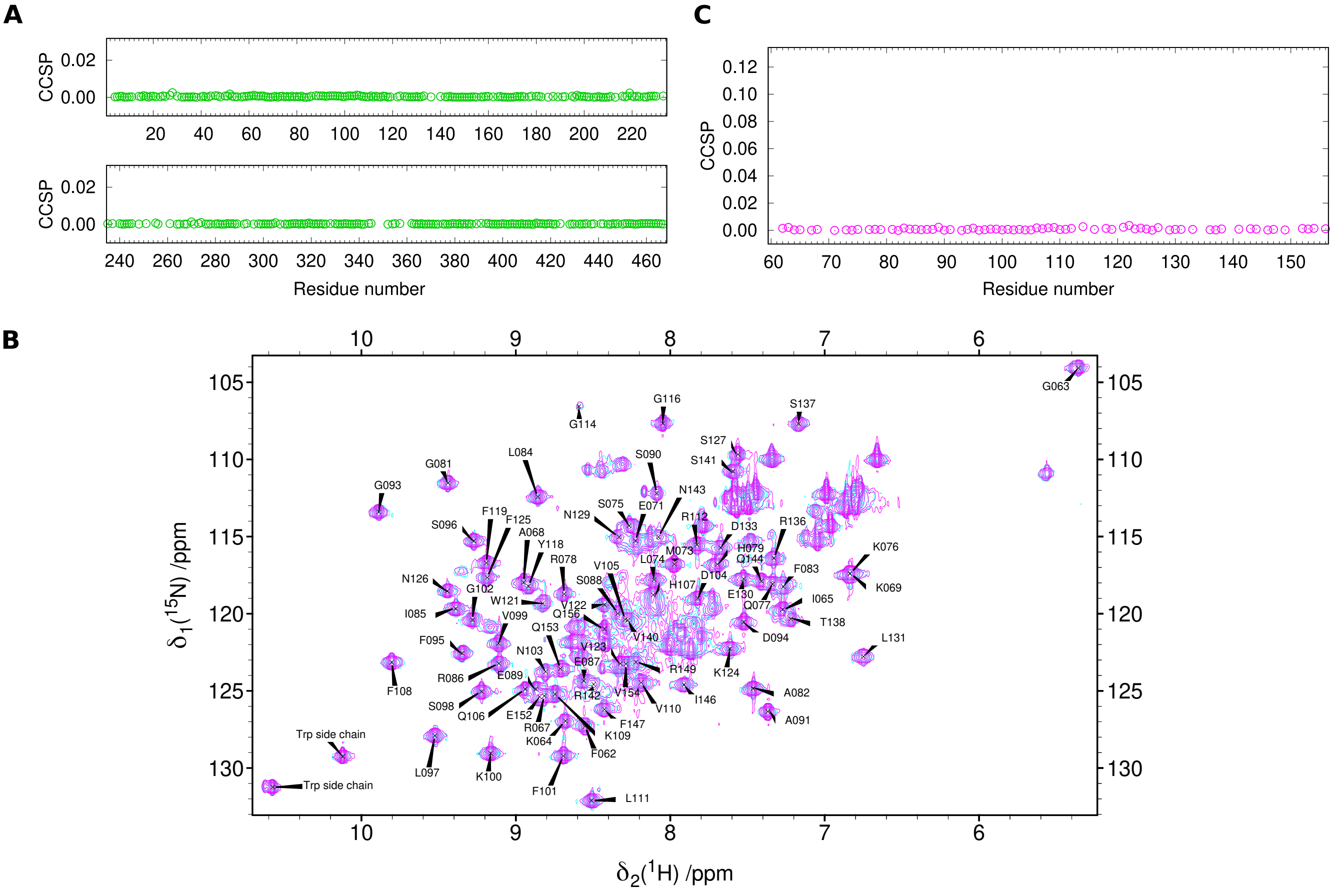

Figure S5. A, CCSP of 100 µM unphosphorylated [^{13}C,^{15}N]-MAP2c upon addition of 100 µM unlabeled Grb2-SH2. B, 2D ^{1}H-^{15}N HSQC spectra of 80 µM [^{15}N]-Grb2-SH2 recorded during titration with unlabeled unphosphorylated MAP2c. C, CCSP of 80 µM [^{15}N]-Grb2-SH2 upon addition of 160 µM unlabeled unphosphorylated MAP2c. Cyan and magenta colors in Panel B correspond to 0 µM and 160 µM MAP2c concentrations, respectively.

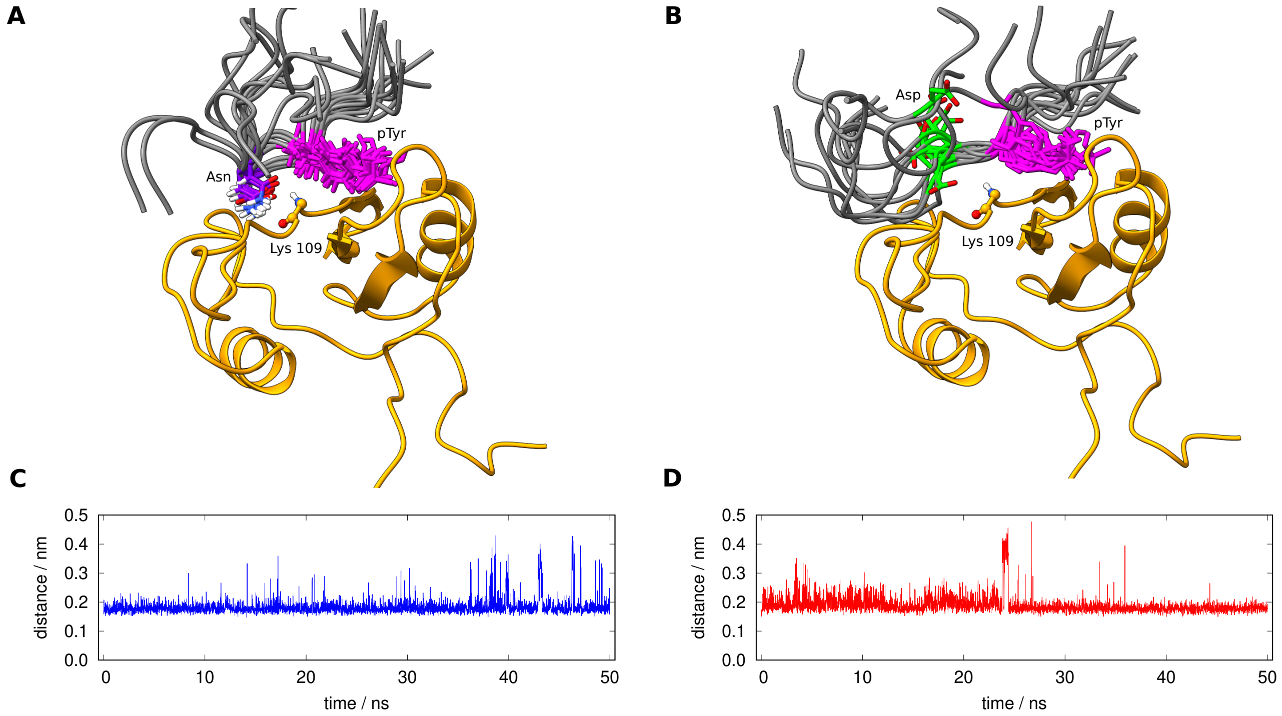

Figure S6. Snapshots of molecular dynamics simulations of phosphopeptides containing the pTyr-Val-Asn-Val sequence (A) and the MAP2c pTyr-Ser-Asp-Thr sequence (B) in complex with Grb2-SH2. Ten snapshots of the phosphopeptide conformations, sampled each 5 ns, are displayed in gray. Only one Grb2-SH2 structure (gold) is shown for the sake of clarity. Residues Asp, Asn, and pTyr of the phosphopeptides are shown as bright green, purple, and magenta sticks, respectively. Nitrogen, oxygen, and hydrogen atoms of the Asn amide and Asp carboxy groups are displayed in blue, red, and white, respectively. Backbone atoms of Grb2-SH2 Lys109, forming a hydrogen bond with the phosphopeptide Asn in the pYXN motif, are displayed in the ball-and-stick representation. Distances between phosphopeptide pTyr and Arg86 of Grb2-SH2 are shown in blue for the the pTyr-Val-Asn-Val sequence (C) and in red for the pTyr-Ser-Asp-Thr sequence (D).

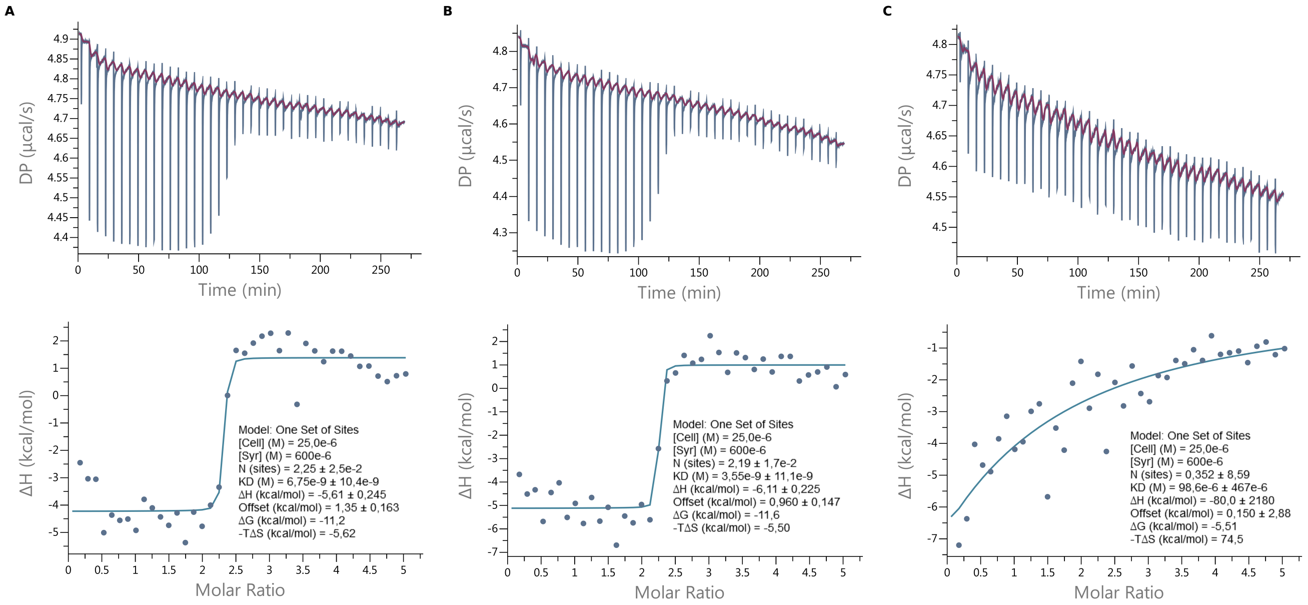

Figure S7. Results of ITC analysis of Fyn-phosphorylated MAP2c titrated by RIIα-PKA in absence (A) and presence of 100 µM Grb2 (B), and a control titration of 100 µM Grb2 by RIIα-PKA (C). The concentration of Fyn-phosphorylated MAP2c in the cell was 25 µM, the concentration of RIIα-PKA and $RIIDD_2$ in the syringe was 600 µM. The obtained binding isotherms in Panel A and B did not differ substantially. Considering the high Grb2 concentration, we can conclude that Grb2 does not interfere with RIIα-PKA binding. The isotherms were steep, resembling titration of unphosphorylated MAP2c by RIIα-PKA (Figure S1A) and did not allow us to estimate a quantitative value of K_D. The control experiment (Panel C) showed that possible interactions between Grb2 and RIIα-PKA are too weak to influence the results.
